# Advanced Pyrrolidine‐Carbamate Self‐Immolative Spacer with Tertiary Amine Handle Induces Superfast Cyclative Drug Release

**DOI:** 10.1002/cmdc.202200279

**Published:** 2022-06-14

**Authors:** Alberto Dal Corso, Margaux Frigoli, Martina Prevosti, Mattia Mason, Raffaella Bucci, Laura Belvisi, Luca Pignataro, Cesare Gennari

**Affiliations:** ^1^ Università degli Studi di Milano Dipartimento di Chimica via C. Golgi, 19 20133 Milan Italy; ^2^ Università degli Studi di Milano Dipartimento di Scienze Farmaceutiche via G. Venezian 21 20133 Milan Italy

**Keywords:** cascade reactions, disassembly, drug delivery, prodrugs, self-immolative spacers

## Abstract

Amine‐carbamate self‐immolative (SI) spacers represent practical and versatile tools in targeted prodrugs, but their slow degradation mechanism limits drug activation at the site of disease. We engineered a pyrrolidine‐carbamate SI spacer with a tertiary amine handle which strongly accelerates the spacer cyclization to give a bicyclic urea and the free hydroxy groups of either cytotoxic (Camptothecin) or immunostimulatory (Resiquimod) drugs. *In silico* conformational analysis and p*K*_a_ calculations suggest a plausible mechanism for the superior efficacy of the advanced SI spacer compared to state‐of‐art analogues.

## Graphical abstract

**The functionalization** of a pyrrolidine‐carbamate self‐immolative spacer with a tertiary amine handle leads to a highly reactive construct, showing fast carbamate cleavage and release of OH‐bearing drugs.

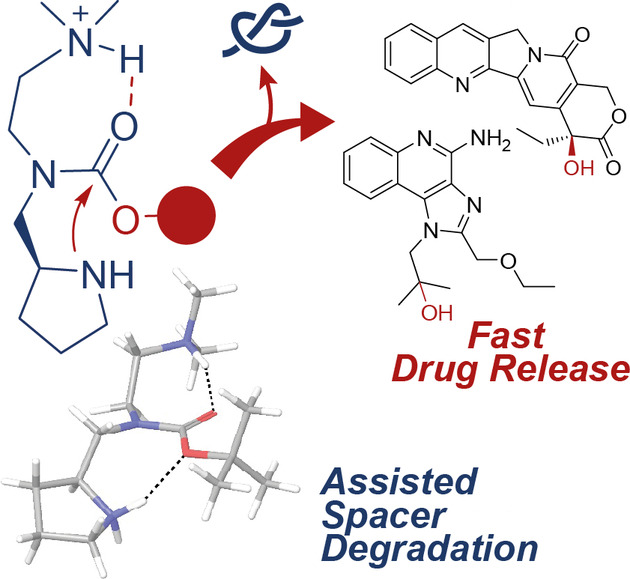

## Introduction

**Figure 1:**
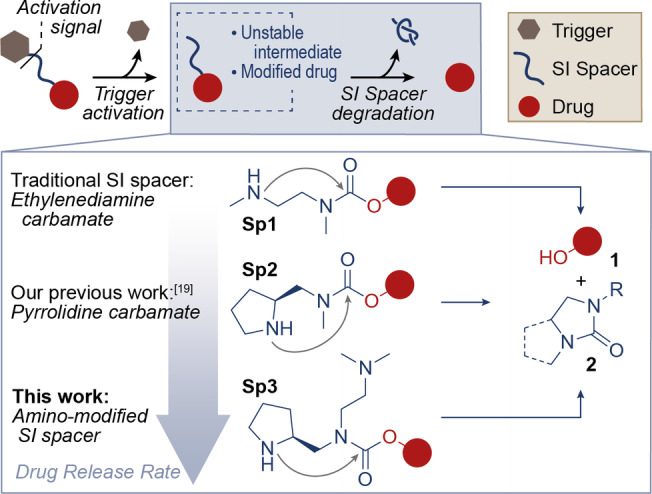
Schematic drug release mechanism of a generic covalent drug conjugate, consisting in the initial trigger activation and subsequent degradation of a self‐immolative (SI) spacer. The molecular structure and degradation mechanism of three cyclizing amine‐carbamate SI spacers (i. e. benchmark ethylenediamine‐carbamate **Sp1**, pyrrolidine‐carbamate **Sp2** ^[19]^ and its engineered derivative **Sp3**, described in this work) is shown: secondary amine cyclization leads to carbamate cleavage, to give the drug's free hydroxy group (**1**) and cyclic urea **2** (mono‐cyclic urea for **Sp1**, bicyclic urea for **Sp2/3**; R=Me for **Sp1/2**, R=(CH_2_)_2_NMe_2_ for **Sp3**). SI spacer cyclization rates increase in the order **Sp1**<**Sp2**<**Sp3**.

The selective delivery of drugs to the site of disease represents a widely pursued research goal, aimed at improving the efficacy and tolerability of pharmacological interventions. In this context, the covalent conjugation of pharmaceutical ingredients to antibodies (resulting in Antibody‐Drug Conjugates, ADCs) represents one of the most validated technologies.^[1]^ Historically, ADCs have been developed to release cytotoxic agents at the tumour site and kill cancer cells selectively, sparing healthy tissues. More recently, the ADC technology was adapted to various classes of pharmaceutical agents, including antibacterial,^[2]^ anti‐inflammatory,^[3]^ and pro‐inflammatory drugs.^[4]^ In addition to antibodies, the covalent drug conjugation to different carriers (e. g. albumin,^[5]^ peptides,^[6]^ small ligands,^[7]^ polymers,^[8]^ etc.) is pushing the boundaries of targeted medicine. In most of these constructs, therapeutic effects are only displayed when the drug is effectively disconnected from the carrier. In this mechanism of action, a key role is played by the so‐called self‐immolative (SI) spacers, i. e. synthetic devices designed to undergo spontaneous disassembly in response to specific stimuli.^[9]^ In particular, different types of activation signals (see “Trigger Activation” in Figure 1) typically lead to the liberation of a reactive functional group in the SI spacer. The latter initiates a variety of intramolecular reactions (mainly electronic cascade in aromatic and π‐extended systems^[10]^ or cyclization of nucleophilic groups)^[11]^ that terminate with the release of thermodynamically‐stable end‐products (see “SI spacer Degradation” in Figure 1).^[12]^ In the prodrug context, not only SI spacers facilitate the drug disconnection from the carrier and its release in a pharmaceutically‐active form, but they also act as chemical adaptors for the whole prodrug assembly.^[13]^ Several conjugation strategies have been proposed to functionalize a variety of bioactive molecules, including amines,^[14]^ phenols,^[15]^ primary^[16]^ and secondary^[17]^ alcohols. In particular, the use of carbamate bonds is a practical and versatile strategy to connect a variety of hydroxy payloads to specific triggers and the ethylenediamine‐carbamate spacer (**Sp1**, Figure 1) has long represented a standard in drug conjugates and other stimuli‐responsive systems.^[18]^

Our group has recently investigated the structural modification of **Sp1**. In 2020, we reported that the pyrrolidine‐carbamate SI spacer **Sp2** (Figure 1) undergoes cyclative cleavage and releases OH‐bearing drugs (**1**) and cyclic urea **2** at higher rates than **Sp1**. The incorporation of **Sp1** and **Sp2** into protease‐activable anticancer prodrugs provided experimental evidence that a fast SI spacer degradation augments the anticancer effects *in vitro*.^[19]^ In light of these data, we speculated that a rapid SI spacer degradation may be particularly important for the prodrug efficacy *in vivo*. Indeed, even if the trigger activation (Figure 1) occurs efficiently and selectively at the site of interest, a long timespan between this initial stimulus and the final drug release prolongs the drug survival in a modified and inactive form. This delayed activation would facilitate the drug migration from the site of disease, its excretion or, at worst, its activation in healthy organs.

We describe herein an advanced SI spacer (**Sp3** in Figure 1) in which a tertiary amine handle enables much faster cyclative drug release compared to previously described spacers of the same class. Thanks to this rapid carbamate cleavage, this accelerated spacer degradation holds promises for improved delivery of therapeutic agents.

## Results and Discussion

**Figure 2:**
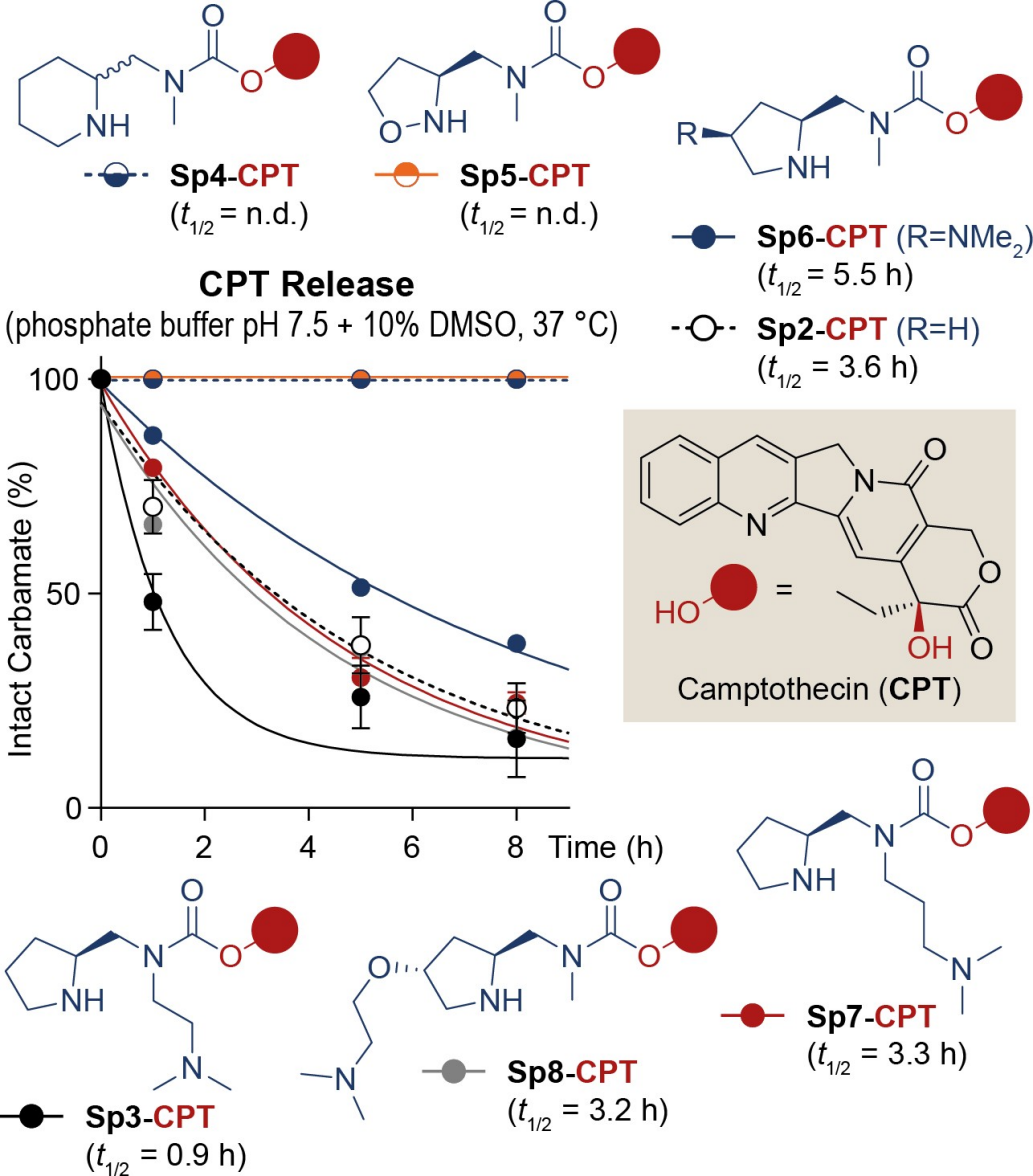
Molecular structure and drug release activity of II‐generation cyclizing SI spacers (**Sp3/8**), connected to the tertiary hydroxy group of Camptothecin (CPT). Experimental procedures for the SI spacer‐CPT module synthesis and release studies are included in the Supporting Information.

We undertook the structural optimization of the pyrrolidine‐carbamate SI spacer **Sp2**, aimed at decreasing the carbamate half‐life (*t*_1/2_) for a faster hydroxy cargo release. The molecular structures of the synthesized SI spacers are reported in Figure 2 (all synthetic procedures are reported in the Supporting Information). Here, the **Sp2** pyrrolidine ring was replaced by either a piperidine (in **Sp4**) or an isoxazolidine (in **Sp5**) cycle. In particular, **Sp4** was designed to assess the impact of the cyclic amine ring size on the SI spacer degradation.^[20]^ On the other hand, the use of an isoxazolidine ring in **Sp5** aimed at evaluating the contribution of the “α‐effect” to the spacer reactivity.^[21]^ While **Sp4** was prepared starting from racemic 2‐piperidinecarbaldehyde, the synthesis of **Sp5** was inspired by Bode's route to 5‐oxaproline.^[22]^ Moreover, as we recently observed the inhibitory effects on the spacer cyclization given by a phosphate monoester group,^[23]^ we devised the SI spacer modification with a basic handle. Since at physiological pH (approximately 7.4) the pyrrolidine is mostly present as protonated species, a second amine handle may force the pyrrolidine deprotonation, thus lowering its p*K*_a_ value and increasing its nucleophilic character.^[24]^ To this end, SI spacers **Sp3** and **Sp6/8** were endowed with a tertiary amine handle connected either at the carbamate N atom (in **Sp3** and **Sp7**) or at the pyrrolidine ring (in **Sp6** and **Sp8**). While the former spacers were rapidly obtained by reductive amination of Boc‐l‐prolinal, the latter were prepared by multistep synthesis, starting from oxygenated proline derivatives, such as 4‐oxo‐l‐proline and hydroxyproline. Firstly, the exocyclic secondary amine of SI spacers **Sp3/8** was conjugated to the tertiary hydroxy group of the anticancer drug Camptothecin (CPT) via carbamate bond. As described recently,[^19^, ^23^] the final spacer‐drug modules **Sp3/8‐CPT** (Figure 2) were isolated as trifluoroacetate salts. These compounds were dissolved in a DMSO/phosphate buffer (pH 7.5) mixture and incubated at 37 °C, followed by aliquot collection at different time points and CPT release analysis by HPLC. The percentage of intact carbamate calculated from peak integrals was plotted versus time, and the spacer cyclization rates were estimated in terms of carbamate half‐life (*t*_1/2_). The results of this first screening are summarized in Figure 2.

Notably, replacement of the native pyrrolidine ring with a piperidine (**Sp4**) and an isoxazolidine (**Sp5**) led to a complete inhibition of the cyclative carbamate cleavage.^[25]^ On the other hand, the SI spacer derivatization with a tertiary amine led to some interesting results in terms of CPT release efficacy. In particular, carbamate **Sp6‐CPT** released CPT with lower rates than reference **Sp2‐CPT** (*t*_1/2_=5.5 and 3.6 h, respectively), while the reactivity of SI spacers **Sp7** (*t*_1/2_=3.3 h) and **Sp8** (*t*_1/2_=3.2 h) was slightly improved, but very similar to the one of native **Sp2**. To our delight, carbamate **Sp3‐CPT** showed a half‐life of 0.9 h, i. e. four times shorter than reference compound **Sp2‐CPT**.^[26]^ All in all, these data indicate that the cyclative cleavage of pyrrolidine‐carbamate SI spacers can be accelerated by a tertiary amine handle, provided that the latter is in close proximity to the electrophilic carbamate bond. Indeed, the tertiary amine installation in a remote position from the carbamate (e. g. in **Sp6** and **Sp8**) showed no significant impact on the CPT release rates compared to reference **Sp2**. The importance of the amine handle proximity is even more evident by considering the better CPT release performance of **Sp3** (where a C‐2 alkyl chain connects the carbamate N atom to the tertiary amino group) compared to **Sp7** (bearing a C‐3 alkyl chain).

With the aim at confirming the superior efficacy of the new SI spacer **Sp3**, we investigated its ability to release a different payload, namely the immunostimulatory drug Resiquimod (R848). This imidazoquinoline (IMD) is a potent agonist of Toll‐like receptors (TLR) 7 and 8, intracellular proteins expressed by several types of immune cells and involved in the host defence from viral infections.^[27]^

**Figure 3:**
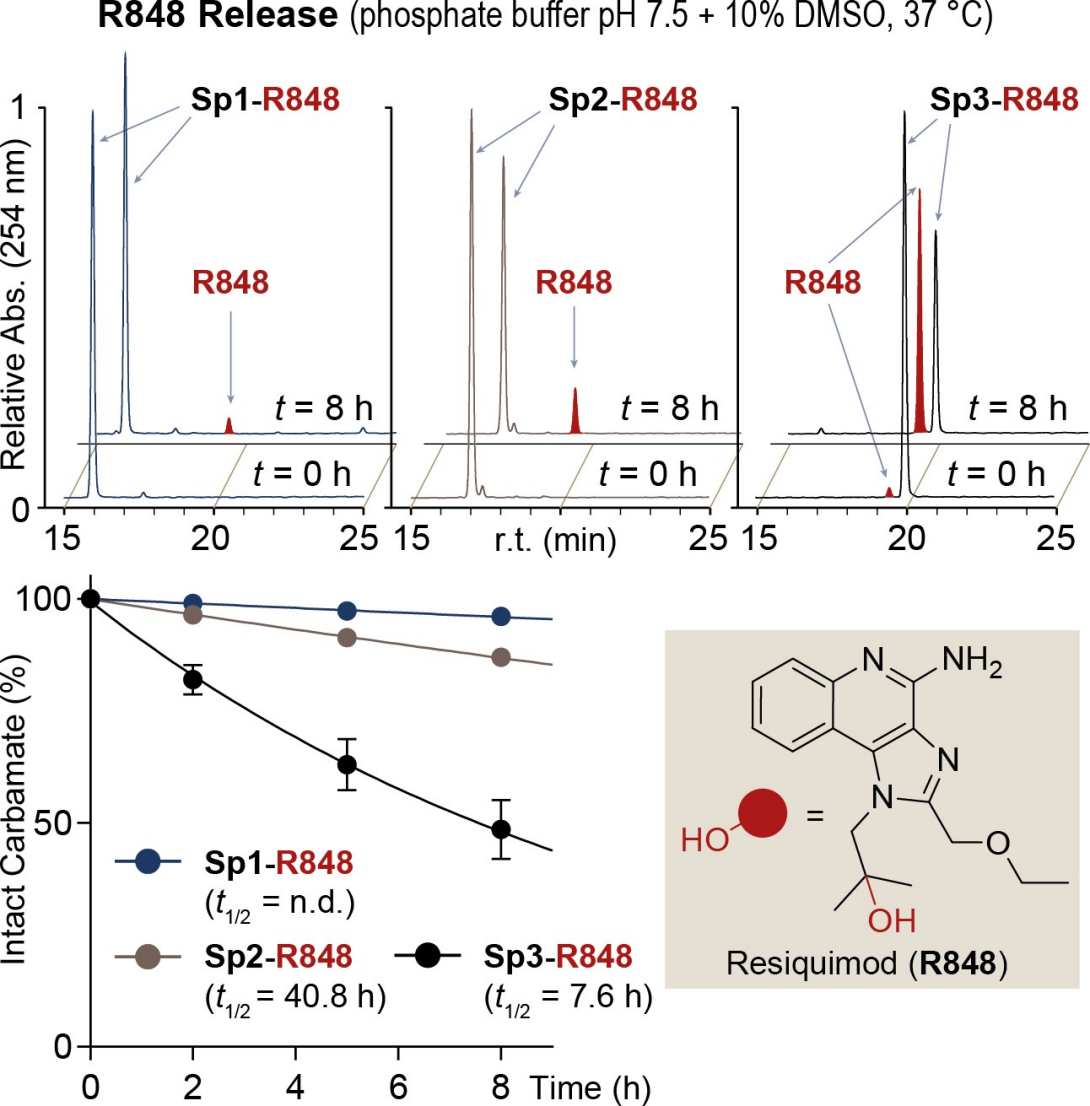
Drug release activity of SI spacers **Sp1/3**, connected to the tertiary hydroxy group of Resiquimod (R848). HPLC traces relative to the stability analysis of carbamates **Sp1/3‐R848** at *t*=0, 8 h are shown (peak of free R848 is highlighted in red) together with stability curves. r.t.: retention time, Abs.: UV absorbance. Experimental procedures for the SI spacer‐R848 carbamate synthesis and release studies are included in the Supporting Information.

By mimicking single‐strand RNA fragments, IMDs induce TLR homodimerization and activate downstream pro‐inflammatory pathways. Due to these pharmaceutical effects, tumour‐targeted IMD prodrugs are being increasingly investigated to selectively activate the immune system at the site of disease, thus improving the outcomes of immunotherapy regimens.[^4^, ^28^] As previously done with CPT, SI spacers **Sp1**, **Sp2** and **Sp3** were connected to the tertiary hydroxy group of R848 via carbamate bond. The resulting **Sp1/3‐R848** adducts were dissolved in a DMSO/phosphate buffer (pH 7.5) mixture and incubated at 37 °C, following drug release analysis as described above. As shown by the HPLC traces and the chart in Figure 3, R848 carbamates proved generally more stable than the analogous CPT constructs. In particular, the native pyrrolidine spacer **Sp2** showed a very slow carbamate cleavage, with a half‐life of 40 h calculated for the **Sp2‐R848** construct. As expected, the drug release activity shown by **Sp2** was superior than the benchmark ethylenediamine‐carbamate spacer **Sp1**, which released only traces of drug after eight‐hour incubation. As observed with the CPT adducts, the tertiary amine‐bearing spacer **Sp3** showed the highest drug release activity of the series, as the half‐life of **Sp3‐R848** carbamate (*t*_1/2_=7.6 h) resulted approximately five times shorter than that of the **Sp2**‐bearing analogue. In this experiment, LC‐MS analysis of the **Sp3‐R848** adduct upon eight‐hour incubation confirmed the formation of bicyclic urea **2** during **Sp3** degradation (see Figure S1 in the Supporting Information). These CPT and R848 release data confirmed the superior performance of the advanced **Sp3** spacer compared to both **Sp2** and, even more dramatically, **Sp1** references.

**Figure 4:**
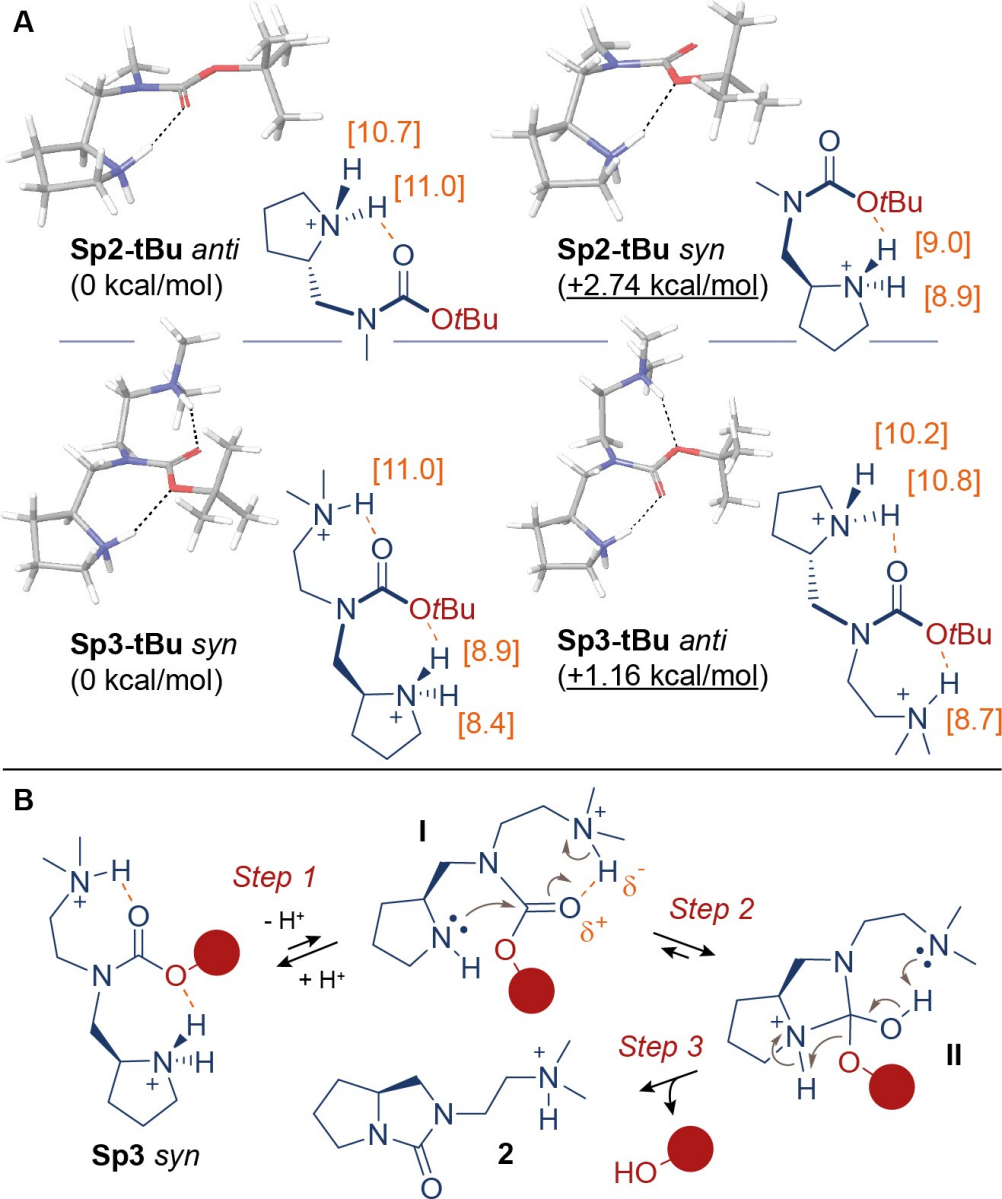
A) Molecular structures of representative conformations for **Sp2/3‐*t*Bu**, optimized at the DFT B3LYP/6‐31G* level. Relative energy differences are calculated from the corresponding solution phase energies (water PBF). Calculated p*K*_a_ values for N‐H^+^ protons are reported (orange). Additional conformations of **Sp2/3‐*t*Bu** are shown in Figure S2 in the Supporting Information). B) Plausible mechanism of carbamate cyclative cleavage carried out by SI spacer **Sp3**.

To qualitatively investigate the structural basis for the **Sp3** spacer exceptional reactivity, we performed conformational analyses by computational methods of both **Sp2** and **Sp3** structures, connected to a generic alcohol (*tert*‐butanol, “*t*Bu”) through a carbamate bond. In particular, Monte Carlo/Energy Minimization (MC/EM) conformational searches^[29]^ were performed at the molecular mechanics level (OPLS3 force field)^[30]^ on carbamates **Sp2‐*t*Bu** and **Sp3‐*t*Bu** in their main ionization state at pH 7.5, corresponding to positively‐charged amino groups (i. e. protonated pyrrolidine in **Sp2‐*t*Bu**, protonated pyrrolidine and tertiary amine in **Sp3‐*t*Bu**). Representative minimum‐energy conformations selected from the molecular mechanics calculations were optimized at the DFT B3LYP/6‐31G* level of theory.^[31]^ Solution phase energies of the obtained stationary points were computed at the same level of theory by single‐point energy calculations including the water/PBF solvent model.^[31]^ Finally, p*K*_a_ values of the protonated species were calculated on DFT minimum energy structures displaying the lowest solution phase energies. According to these calculations, carbamate **Sp2‐*t*Bu** adopts a preferential conformation (referred to as “Sp2‐*t*Bu *anti*” in Figure 4A) in which the pyrrolidinium ion engages the carbonyl sp^2^‐hybridized O atom in a hydrogen bond, forming a seven‐membered ring. On the other hand, a similar intramolecular interaction of the pyrrolidinium ion with sp^3^‐hybridized O atom leads to the “Sp2‐*t*Bu *syn*” conformation, 2.74 kcal/mol higher in energy than the “a*nti”* counterpart.

“*Anti*” rotamers in carbamates are typically favored over the “*syn*” counterparts by 1.0–1.5 kcal/mol,^[32]^ and the 2.74 kcal/mol increased stability of “Sp2‐*t*Bu *anti*” versus “Sp2‐*t*Bu *syn*” indicates that rotational equilibrium of amine‐carbamate modules can be dramatically influenced by intramolecular H bonding. Concerning **Sp3‐*t*Bu**, two simultaneous H bonds can be formed by two donors (i. e. pyrrolidinium and trialkyl ammonium ions), which can individually engage either O atoms (Figure 4A). In contrast to the **Sp2‐*t*Bu** data, the preferred **Sp3‐*t*Bu** conformation features the pyrrolidinium ion engaging the sp^3^‐hybridized O atom, whereas the sp^2^‐hybridized O atom binds the trialkyl ammonium ion. This “Sp3‐*t*Bu *syn*” conformation is favored by 1.16 kcal/mol over the “Sp3‐*t*Bu *anti*” counterpart, in which the pyrrolidinium and trialkyl ammonium ions engage the sp^2^‐ and sp^3^‐hybridized O atoms, respectively (Figure 4, for additional conformations of **Sp2‐*t*Bu** and **Sp3‐*t*Bu**, see Figure S2 in the Supporting Information). Interestingly, the two pyrrolidinium protons in the “Sp3‐*t*Bu *syn*” conformation proved more acidic (calculated p*K*_a_=8.9, 8.4) than the trialkyl ammonium species (p*K*_a_=11.0).

These *in silico* data suggest a possible explanation for the observed SI spacer reactivity. Firstly, at the onset of SI spacer degradation, it is reasonable to assume that the nucleophilic attack of the uncharged pyrrolidine N atom to the carbonyl group occurs with a N−C−O bond angle >90°, following the well‐known Bürgi‐Dunitz trajectory.^[33]^ The three‐dimensional analysis of the Sp‐carbamate modules indicates that this geometry of attack is only accessible by the carbamate *syn* rotamers. Secondly, considering the perturbed p*K*_a_ of the pyrrolidinium ion in the “Sp3‐*t*Bu *syn*” structure, it is possible that the pyrrolidine nucleophilic attack in **Sp3** is also facilitated by a preferential proton dissociation in aqueous medium.

In summary, the tertiary amine proximity to the carbamate group may facilitate the SI spacer degradation at different stages of the cyclization reaction, following the mechanism proposed in Figure 4B. In particular, upon pyrrolidine deprotonation (Step 1, equilibrium governed by the pH of the aqueous medium), the intramolecular hydrogen bond between the trialkylammonium ion and the sp^2^‐hybridized O atom in intermediate I may facilitate the pyrrolidine cyclization (Step 2) to give the tetrahedral intermediate II. Here, the basicity of the tertiary amine may favor the restoration of the sp^2^‐hybridized C center (bicyclic urea formation) and the liberation of the hydroxy group (Step 3). This impact of neighbouring groups on bond cleavage kinetics is reminiscent of “catalytic triads” in the active site of hydrolytic enzymes,^[34]^ which have inspired the development of synthetic enzyme mimics.^[35]^ Similarly, a positively‐charged lysine ϵ‐amine group in an ADC construct was recently found to act as acid‐catalyst, and exploited to induce modifications of acetal and succinimide labels connected to the antibody core.^[36]^

## Conclusions

The present work describes a highly reactive pyrrolidine‐carbamate SI spacer (**Sp3**) in which a superfast carbamate cyclative cleavage is induced by the presence of a tertiary amine handle in close proximity to the carbamate bond. This advanced spacer showed better performance than **Sp2** in the release of both the cytotoxic agent CPT and immunostimulatory drug R848. *In silico* conformational analysis and p*K*_a_ calculations allowed us to propose a plausible rationale for the superior efficacy of the advanced SI spacer compared to reference **Sp2**. Considering the very slow R848 release observed with state‐of‐art SI spacers **Sp1** and **Sp2**, the new spacer **Sp3** may be pivotal for the design of cleavable R848 conjugates, alternative to previous strategies for IMD derivatization.^[37]^ Moreover, as we reported for the **Sp2** reference,[^19^, ^23^] the **Sp3** module can be easily installed into functional drug delivery systems. For instance, the **Sp3** connection to a *para*‐aminobenzyl carbamate (PABC) spacer will access to protease‐activable prodrugs, which represent the backbone of drug delivery technologies, including marketed ADCs.^[38]^ In general, **Sp3** application in different types of stimuli‐responsive materials can be envisioned as a valid alternative to current strategies for hydroxyl cargo delivery.[^13^, ^16^, ^17^]

## Conflict of interest

The authors declare no conflict of interest.

1

## Supporting information

As a service to our authors and readers, this journal provides supporting information supplied by the authors. Such materials are peer reviewed and may be re‐organized for online delivery, but are not copy‐edited or typeset. Technical support issues arising from supporting information (other than missing files) should be addressed to the authors.

Supporting Information

## Data Availability

The data that support the findings of this study are available in the supplementary material of this article.
