# Supplementary material for: Advanced Pyrrolidine‐Carbamate Self‐Immolative Spacer with Tertiary Amine Handle Induces Superfast Cyclative Drug Release
Source: ChemMedChem. 2022 Jun 14;17(15):e202200279. doi: 10.1002/cmdc.202200279 (PMC9544318; doi:10.1002/cmdc.202200279)

ChemMedChem

Supporting Information

Advanced Pyrrolidine-Carbamate Self-Immolative Spacer with Tertiary Amine Handle Induces Superfast Cyclative Drug Release

Alberto Dal Corso,* Margaux Frigoli, Martina Prevosti, Mattia Mason, Raffaella Bucci, Laura Belvisi, Luca Pignataro, and Cesare Gennari*

Supporting Information

Table of Contents

Supplementary Figures **S2**

Materials and Methods **S3**

List of Abbreviations and Symbols **S4**

Synthetic procedures **S5**

General Procedures S5

Synthesis of Sp3-CPT S6

Synthesis of Sp4-CPT S7

Synthesis of Sp5-CPT S8

Synthesis of Sp6-CPT S10

Synthesis of Sp7-CPT S13

Synthesis of Sp8-CPT S14

Synthesis of Sp1-R848, Sp2-R848, Sp3-R848 S19

Carbamate Cleavage Studies **S24**

Experimental Procedure for Sp-CPT Modules S24

Experimental Procedure for Sp-R848 modules S25

Computational Studies **S26**

Appendix **S28**

HPLC Data – Sp-CPT Modules S28

HPLC Data – Sp-R848 Modules S35

NMR Spectra S38

Supplementary Figures

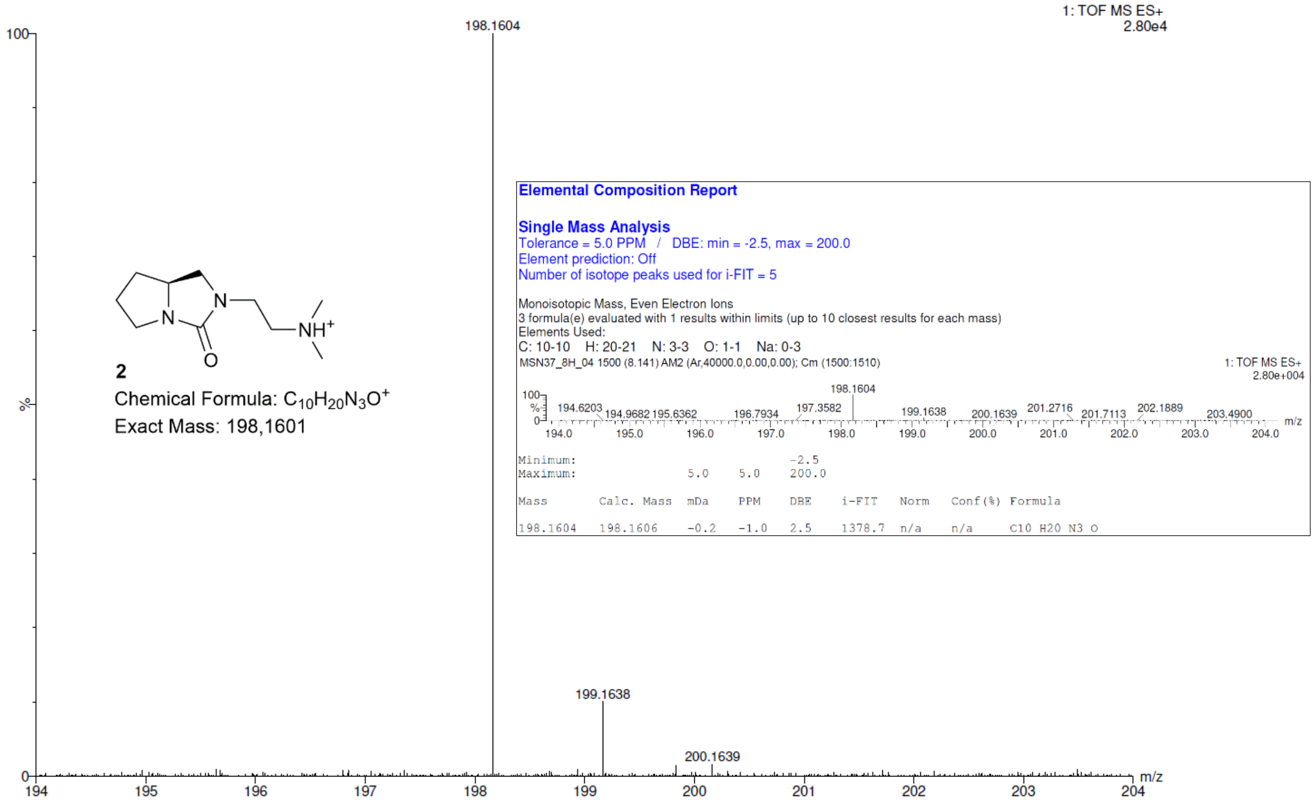

Figure S1. HRMS spectrum and elemental composition report for bicyclic urea **2** (**Sp3** cyclization end-product) detected by LC-MS analysis of **Sp3-R848** upon incubation for 8 h in phosphate buffer.

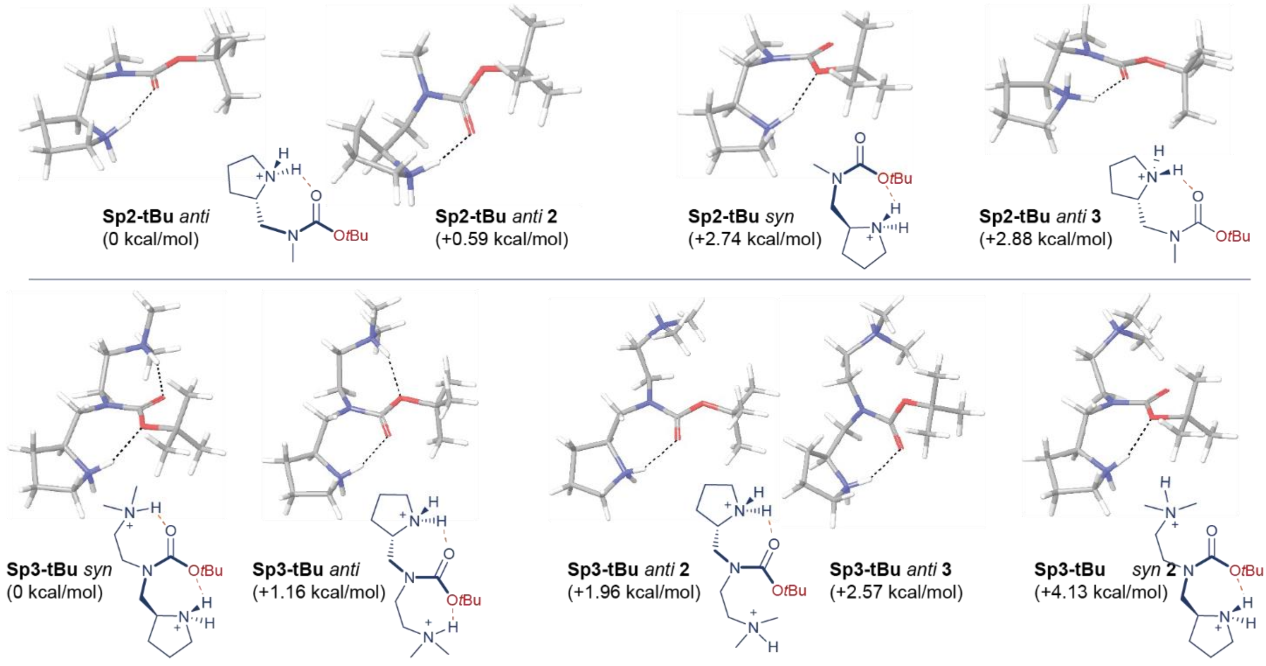

Figure S2. Molecular structures of representative conformations for **Sp2/3-tBu**, optimized at the DFT B3LYP/6-31G* level. Relative energy differences are calculated from the corresponding solution phase energies (water PBF).

Materials and Methods

All manipulations requiring anhydrous conditions were carried out in flame-dried glassware, with magnetic stirring and under a nitrogen atmosphere. All commercially available reagents were used as received. Anhydrous solvents were purchased from commercial sources and withdrawn from the container by syringe, under a slight positive pressure of nitrogen. The reactions were monitored by analytical thin-layer chromatography (TLC) using silica gel 60 F254 pre-coated glass plates (0.25 mm thickness). Visualization was accomplished by irradiation with a UV lamp and/or staining with a ceric ammonium molybdate solution, 2,4-dinitrophenylhydrazine, concentrated H_2SO_4 or ninhydrin. Flash column chromatography was performed according to the method of Still and co-workers[1] using Chromagel 60 ACC (40-63 µm) silica gel. Proton chemical shifts are reported in ppm (δ) with the solvent reference relative to tetramethylsilane (TMS) employed as the internal standard ($CDCl_3$ δ = 7.26 ppm; CD_2Cl_2, δ = 5.32 ppm; d_6-DMSO, δ = 2.50 ppm; CD_3OD, δ = 3.33 ppm, d_8-THF δ = 3.58 ppm, 1.73 ppm). The following abbreviations are used to describe spin multiplicity: s = singlet, d = doublet, t = triplet, q = quartet, m = multiplet, bs = broad signal, dd = doublet of doublet. Carbon NMR spectra were recorded on a spectrometer operating at 100.63 MHz, with complete proton decoupling. Carbon chemical shifts are reported in ppm (δ) relative to TMS with the respective solvent resonance as the internal standard ($CDCl_3$, δ = 77.16 ppm; CD_2Cl_2, δ = 54.00 ppm; d_6-DMSO, δ = 39.51 ppm; CD_3OD, δ = 49.05 ppm; d_8-THF δ = 67.57 ppm, 25.37 ppm). HPLC purifications were performed on Dionex Ultimate 3000 equipped with Dionex RS Variable Wavelenght Detector (column: Atlantis Prep T3 OBDTM 5 µm 19 x 100 mm; flow 10 ml/min unless stated otherwise). HPLC analysis of carbamate stability was performed on a Waters 515 HPLC pumps equipped with 996 photodiode array detector and Waters Atlantis T3 - 5 µm - 4.6 x 100 mm column (injection volume: 200 µL) and on a Jasco LC-4000 HPLC System equipped with MD-4010 photodiode array detector and Phenomenex Gemini-NX 5 µm - 4.6 x 150 mm column (injection volume: 20 µL). High-resolution mass spectrometry analysis (HRMS, 4 decimal places) were performed on a Q-TOF Synapt G2-Si instrument available at the MS facility of the Unitech COSPECT at the University of Milan. Low resolution mass spectra (MS, 1 and 2 decimal places) were recorded on a Thermo Scientific LCQ Fleet Ion Trap Mass Spectrometer (ESI source). Camptothecin carbonate **CPT-PNP**[2], Boc-Hyp-OMe,[3] and **Sp2-CPT**[4] were prepared following published procedures.

---

1 W. C. Still, M. Kahn, A. Mitra, *J. Org. Chem.* 1978, **43**, 2923.

2 E. Riva, D. Comi, S. Borrelli, F. Colombo, B. Danieli, J. Borlak, L. Evensen, J. B. Lorens, G. Fontana, O. M. Gia, L. Dalla Via D. Passarella, *Bioorg. Med. Chem.* 2010, **18**, 8660.

3 K. K. Schumacher, J. Jiang, M. M. Joullié, *Tetrahedron: Asymmetry* 1998, **17**, 47.

4 A. Dal Corso, V. Borlandelli, C. Corno, P. Perego, L. Belvisi, L. Pignataro, C. Gennari, *Angew. Chem. Int. Ed.* 2020, **59**, 4176.

List of Abbreviations and Symbols

AcOEt	Ethyl Acetate	Me	Methyl
AcOH	Acetic acid	MeCN	Acetonitrile
aq.	Aqueous solution	MeOH	Methanol
Boc	Tert-butyloxycarbonyl-	min.	Minutes
DIBAL-H	Diisobutylaluminum hydride	MS	Mass Spectroscopy
DIPEA	*N,N*-Diisopropylethylamine	MW	Molecular weight
DMAP	4-Dimethylaminopyridine	NMR	Nuclear Magnetic Resonance
DMF	Dimethylformamide	NMO	4-Methylmorpholine N-oxide
DMP	Dess-Martin periodinane	ppm	Part per million
DMSO	Dimethylsulfoxide	r.t.	Room temperature
equiv.	Equivalents	R_f	Retention factor
ESI	Electrospray ionization	sat.	Saturated
Et	Ethyl	*t*Bu	*tert*-Butyl
h	Hours	*tert*	Tertiary
Hex	*n*-Hexane	TEA	Triethylamine
HPLC	High performance liquid chromatography	TFA	Trifluoroacetic acid
HRMS	High resolution mass spectroscopy	THF	Tetrahydrofuran
Hyp	(2*S*,4*R*)-4-Hydroxyproline	t_R	Retention time
J	Scalar coupling constants	δ	Chemical shift
KHMDS	Potassium hexamethyldisilazide		

Synthetic procedures

General Procedures

General procedure A for partial reduction of L-Proline esters. Ester (1 equiv.) was dissolved in dry CH_2Cl_2 (0.2 M), stirred under nitrogen atmosphere, and cooled at -78 °C with an acetone/dry ice bath. DIBAL-H (1 M in hexane, 1 equiv.) was slowly added to the solution and the mixture was stirred at -78 °C until starting material consumption was detected by TLC. Later on, MeOH was added at -78 °C (3 x 30 µL) and the solution was stirred for 5 minutes. The mixture was warmed to r.t. and transferred into a separatory funnel containing a sat. aqueous NH_4Cl solution (25 mL). The solution was extracted with CH_2Cl_2 (3 x 30 mL), dried and concentrated in vacuum. Unless stated otherwise, the resulting aldehyde was used in the following synthetic step without further purification.

General procedure B for reductive amination. Aldehyde (1 equiv.) was dissolved in CH_2Cl_2/AcOH under nitrogen atmosphere. Primary amine (4 equiv.) and $NaBH(OAc)_3$ (5 equiv.) were added to the aldehyde solution and the reaction was stirred at r.t. overnight. The mixture was transferred into a separatory funnel and diluted with CH_2Cl_2 (10 mL). A 10% Na_2CO_3 solution (20 mL) was added and the stirring mixture was flushed with nitrogen. NaOH 2 M was then added until pH ~12. The mixture was extracted with CH_2Cl_2 (3 x 25 mL). Collected organic phases were washed with brine (1 x 5 mL), dried and concentrated under vacuum.

General procedure C for Boc deprotection. To an ice-cold CH_2Cl_2 solution of the *N*-Boc-protected compound, half volume of TFA was added added dropwise at 0 °C and the mixture was stirred at r.t. for 1 h. The solvent was evaporated and then CH_2Cl_2 was added for two times to the residue followed by evaporation under vacuum, to afford the amine TFA salt.

Synthesis of Sp3-CPT

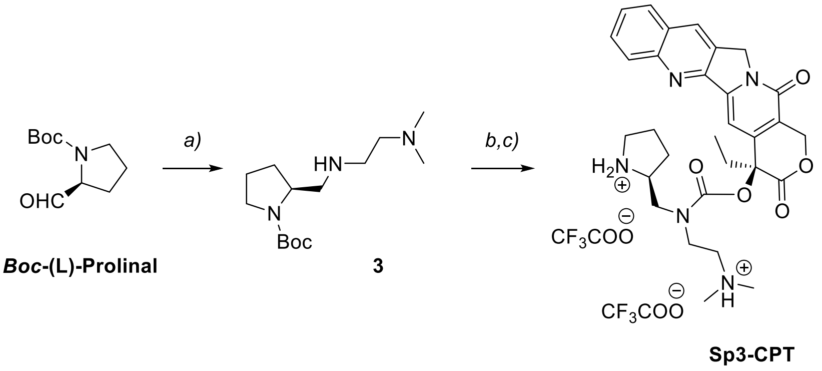

Scheme S1. REAGENTS AND CONDITIONS: *a) N,N*-dimethylethylenediamine, NaBH(OAc)$_3$, AcOH in dry CH$_2$Cl$_2$, r.t., overnight; *b)* **CPT-PNP**, dry CH$_2$Cl$_2$, r.t. 3h;. *c)* TFA/CH$_2$Cl$_2$ 1:2, 0 °C to r.t., 45 min.

Tert-butyl (S)-2-(((2-(dimethylamino)ethyl)amino)methyl)pyrrolidine-1-carboxylate (**3**)

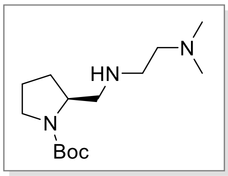

$C_{14}H_{29}N_3O_2$
MW: 271.41 g • mol^{-1}

Boc-(L)-Prolinal (186 µL, 1 mmol, 1 equiv.) was dissolved in CH$_2$Cl$_2$/AcOH (20 + 2 mL) under nitrogen atmosphere and treated with *N,N*-dimethylethylenediamine (95% wt, 438 µL, 4 mmol, 4 equiv.) following General Procedure B. The crude product was purified with column chromatography (gradient from 10% to 20% MeOH in CH$_2$Cl$_2$) to give amine **3** (232 mg, quant) as a colourless oil.

^{1}H NMR (400 MHz, MeOD) δ 3.89 (bs, 1H), 3.39-3.28 (m, 2H), 2.87-2.67 (m, 3H), 2.59 (m, 1H), 2.53-2.41 (m, 2H), 2.27 (s, 6H), 2.04-1.77 (m, 4H), 1.47 (s, 9H) ppm; MS (ESI): *m/z* calcd. for $[C_{14}H_{29}N_3O_2]^+$: 272.23 [*M*+H]$^+$, found: 272.29.

Sp3-CPT

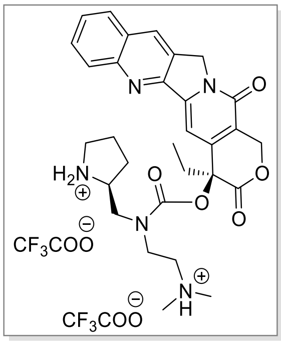

$C_{30}H_{35}N_5O_5$
MW: 545.64 g • mol^{-1} + TFA

To a solution of **CPT-PNP** (9 mg, 17 µmol, 1 equiv.) in dry CH$_2$Cl$_2$ was added compound **3** (13.6 mg, 50 µmol, 3 equiv.) under nitrogen atmosphere followed by DIPEA (11.9 µL, 68 µmol, 4 equiv.). The reaction

was stirred at 25 °C for 2h. The crude product [R_f = 0.40 (9:1 CH_2Cl_2/MeOH)] was dissolved in dry CH_2Cl_2 (600 µL, 0.05 M) and treated following General Procedure C. The crude product was then purified by HPLC (eluent A: H_2O + 0.1% TFA; eluent B: MeCN, ramp from 0% B (at min 1) to 60% B (at min 10.5), t_R (product): 8.6 min). The purified product was lyophilized to give carbamate **Sp3-CPT** as a yellow solid (22 mg, 95%).

MS (ESI): *m/z* calcd. for $[C_{30}H_{35}N_5O_5]^+$: 546.27 $[M+H]^+$, found: 546.48; HRMS (ESI) *m/z* calcd. for $[C_{30}H_{35}N_5O_5]^+$: 546.2716 $[M+H]^+$, found: 546.2722.

Synthesis of Sp4-CPT

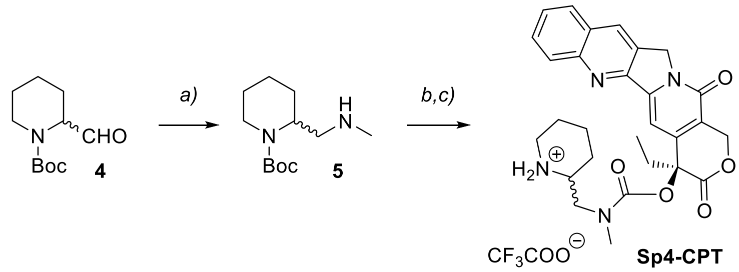

Scheme S2. REAGENTS AND CONDITIONS: *a)* $MeNH_2$, $NaBH(OAc)_3$ in dry CH_2Cl_2, r.t., overnight; b) **CPT-PNP**, dry CH_2Cl_2, r.t. 3h; c) TFA/CH_2Cl_2 1:2, 0 °C to r.t., 45 min.

Tert-butyl 2-((methylamino)methyl)piperidine-1-carboxylate (**5**)

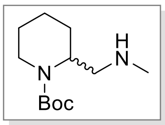

$C_{12}H_{24}N_2O_2$
MW: 228.34 g • mol^{-1}

1-*N-Boc*-2-piperidinecarbaldehyde (**4**) (racemate, 214 mg, 1 mmol, 1 equiv.) was dissolved in CH_2Cl_2/AcOH (20 + 2 mL) under nitrogen atmosphere and treated with methylamine (33% wt in absolute ethanol, 373.6 µL, 3 mmol, 3 equiv.) following General Procedure B. Amine **5** was obtained as a colourless oil (100 mg, 45%).

^{1}H NMR (400 MHz, MeOD) δ 4.35 (m, 1H), 3.96 (m, 1H), 2.88-2.82 (m, 2H), 2.64 (dd, J = 12.5, 6.6 Hz, 1H), 2.39 (s, 3H), 1.70-1.56 (m, 6H), 1.46 (s, 9H) ppm.

Sp4-CPT

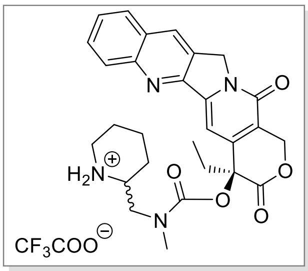

$C_{28}H_{30}N_4O_5$
MW: 502.57 g • mol^{-1} + TFA

To a solution of **CPT-PNP** (20 mg, 40 μmol, 1 equiv.) in dry CH_2Cl_2 was added compound **5** (36 mg, 155 μmol, 4 equiv.) under nitrogen atmosphere. The mixture was stirred at 20 °C overnight. The crude product [R_f = 0.83 (9:1 CH_2Cl_2/MeOH) mixture of diastereoisomers.] was dissolved in dry CH_2Cl_2 (800 µL, 0.05 M) and treated following General Procedure C. The crude product was then purified by HPLC (eluent A: H_2O + 0.1% TFA; eluent B: MeCN, ramp from 0% B (at min 1) to 60% B (at min 10.5), t_R (product): 9.8 min). The product was isolated as a mixture of diastereoisomers and lyophilized to give **Sp4-CPT** as a yellow solid (22 mg, 89% over two steps).

MS (ESI): *m/z* calcd. for $[C_{28}H_{30}N_4O_5]^+$: 503.23 [*M*+H]+, found: 503.37; HRMS (ESI) *m/z* calcd. for $[C_{28}H_{30}N_4O_5]^+$: 503.2294 [*M*+H]+, found: 503.2302.

Synthesis of Sp5-CPT

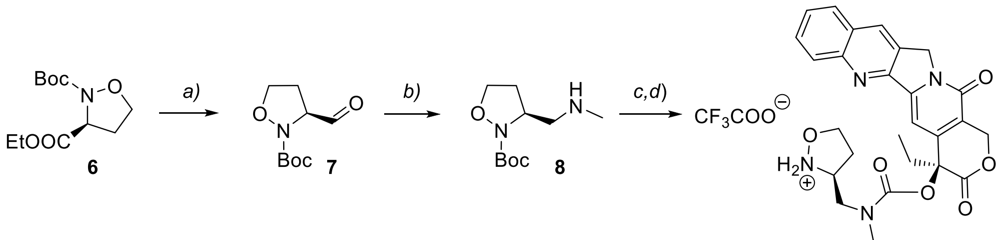

Sp5-CPT

Scheme S3. REAGENTS AND CONDITIONS: *a)* 1 M DIBAL-H in Hexane, dry CH_2Cl_2, -78 °C, 2h; *b*) $MeNH_2$, $NaBH(OAc)_3$, dry CH_2Cl_2, overnight; *c*) **CPT-PNP**, dry CH_2Cl_2, r.t. 3h; *d*) TFA/CH_2Cl_2 1:2, 0 °C to r.t., 45 min.

Tert-butyl (S)-3-formylisoxazolidine-2-carboxylate (**7**)

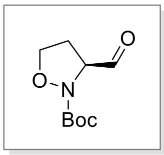

$C_9H_{15}NO_4$
MW: 201.22 g • mol^{-1}

Ethyl ester **6**[5] (150 mg, 611 μmol, 1 equiv.) was dissolved in CH_2Cl_2 (0.2 M) and treated following General Procedure A. Crude product was purified by column chromatography (CH_2Cl_2/MeOH, MeOH gradient from 2% to 5%). Final aldehyde **7** was obtained as a colourless oil (56 mg, 46%).

R_f = 0.35 (1:1 EtOAc/Hex, stained with ninhydrin); ^{1}H NMR (400 MHz, $CDCl_3$) δ 9.61 (d, *J* = 1.4 Hz, 1H), 4.59 (dd, *J* = 9.1, 4.6 Hz, 1H), 4.07 (m, 1H), 3.78 (dd, *J* = 16.3, 9.1 Hz, 1H), 2.58-2.38 (m, 2H), 1.52 (s, 9H) ppm.

5 C.E. Murar, T. J. Harmand, J. W. Bode, *Bioorg. Med. Chem.* 2017, **25**, 4996.

O-acetyl-N-(((S)-2-(tert-butoxycarbonyl)isoxazolidin-3-yl)methyl)-N-methylhydroxylammonium (**8**)

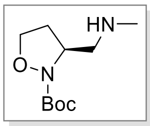

$C_{10}H_{20}N_2O_3$
MW: 216.28 g • mol^{-1}

Aldehyde **7** (56 mg, 278 µmol, 1 equiv.) was dissolved in a 10% mixture of AcOH in CH_2Cl_2 (556 µL in 5.56 mL CH_2Cl_2) under nitrogen atmosphere and treated with methylamine (33% wt in absolute ethanol, 138 µL, 1.11 mmol, 4 equiv.) and a reducing agent following General Procedure B. The crude product was purified with column chromatography (gradient from 10 to 20% MeOH in CH_2Cl_2), to give amine **8** (22 mg, 30%) as an oil.

R_f = 0.38 (15% MeOH in CH_2Cl_2 + 0.2% TEA, stained with ninhydrin); ^{1}H NMR (400 MHz, MeOD) δ 4.53 (m, 1H), 4.12 (m, 1H), 3.69 (m, 1H), 3.10 (dd, J = 12.9, 3.7 Hz, 1H), 2.99 (dd, J = 12.9, 10.4 Hz , 1H), 2.71 (s, 3H), 2.54 (m, 1H), 1.99 (m, 1H), 1.51 (s, 9H) ppm.

Sp5-CPT

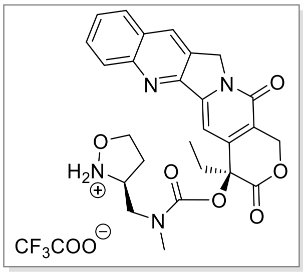

$C_{26}H_{26}N_4O_6$
MW: 490.52 g • mol^{-1} + TFA

To a solution of **CPT-PNP** (7 mg, 14 µmol, 1 equiv.) in dry CH_2Cl_2 was added compound **8** (14 mg, 49 µmol, 3.5 equiv.) under nitrogen atmosphere followed by DIPEA (10 µL, 56 µmol, 4 equiv.). The reaction was stirred at 20 °C for 2 h. The crude product [R_f = 0.24 (9:1 CH_2Cl_2/MeOH); MS (ESI): *m/z* calcd. for $[C_{31}H_{34}N_4O_8]^+$: 591.24 [*M*+H]$^+$; found: 590.20.] was dissolved in dry CH_2Cl_2 (800 µL, 0.05 M) and treated with TFA following General Procedure C. The crude product was then purified by HPLC (eluent A: H_2O + 0.1% TFA; eluent B: MeCN, ramp from 10% B (at min 1) to 57% B (at min 10), t_R (product): 9.0 min). The purified product was lyophilized to give **Sp5-CPT** as a yellow solid (20 mg, 83% over two steps).

MS (ESI): *m/z* calcd. for $[C_{26}H_{26}N_4O_6]^+$: 513.17 [*M*+Na]$^+$, found: 513.20; HRMS (ESI) *m/z* calcd.. for $[C_{26}H_{26}N_4O_6]^+$: 491.1931 [*M*+H]$^+$, found: 491.1933, *m/z* calcd. for $[C_{26}H_{26}N_4O_6]^+$: 513.1750 [*M*+Na]$^+$, found: 513.1752.

Synthesis of Sp6-CPT

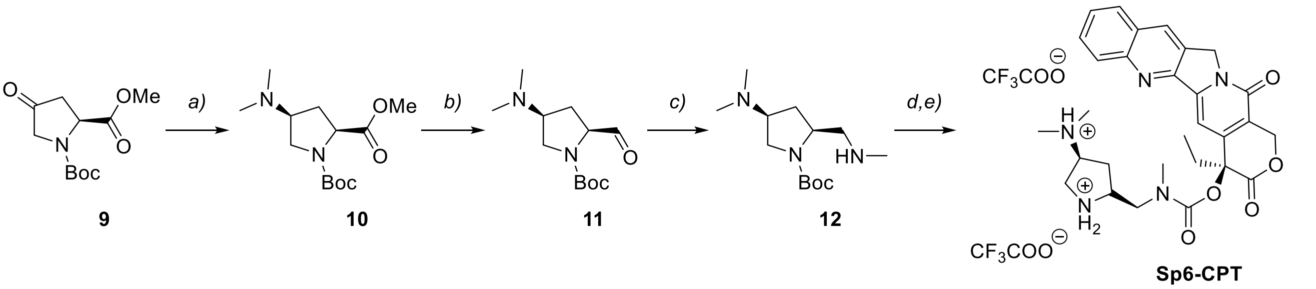

Scheme S4. REAGENTS AND CONDITIONS: *a*) $HNMe_2$, $NaBH_3CN$, AcOH in THF, 40 °C, 3h; *b*) DIBAL-H, dry CH_2Cl_2, -80 °C, 2h; *c*) $MeNH_2$, $NaBH(OAc)_3$, AcOH in dry CH_2Cl_2, r.t. 6h; *d*) **CPT-PNP** in dry CH_2Cl_2, r.t. 3h; *e*) TFA/CH_2Cl_2 1:2, 0 °C to r.t., 45 min.

1-(tert-butyl) 2-methyl (2S,4S)-4-(dimethylamino)pyrrolidine-1,2-dicarboxylate (**10**):

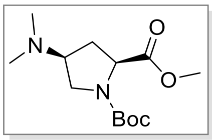

$C_{13}H_{24}N_2O_4$
MW: 272.35 g • mol^{-1}

To a stirred solution of ketone **9** (150 mg, 620 µmol, 1 equiv.) in dry THF (4.5 mL) dimethylamine (2.0 M in MeOH, 1.24 mL, 2.48 mmol, 4 equiv.) and acetic acid (53 µL, 930 µmol, 1.5 equiv.) were added under nitrogen atmosphere. $NaBH_3CN$ (117 mg, 1.86 mmol, 3 equiv.) was added and the mixture was stirred at r.t. for 2 h. THF was evaporated in vacuo. The crude solid was dissolved in ethyl acetate (50 mL) and transferred into a separatory funnel. A saturated aqueous sodium bicarbonate (20 mL) was added, followed by layer separation. The organic phase was then washed with brine (10 mL), dried and concentrated. The solid crude was purified with flash chromatography (gradient: from 1% to 4% MeOH in CH_2Cl_2) to obtain the final product as a yellow oil (156 mg, 93%).

R_f = 0.40 (9:1 CH_2Cl_2/MeOH); ^{1}H NMR (400 MHz, MeOD) δ 4.31 (m, 1H), 3.91 (dd, J = 10.0, 7.7 Hz, 1H), 3.82 (m, 1H), 3.77 (bs, 3H), 3.53 (m, 1H), 2.73-2.63 (m, 7H), 2.18 (m, 1H), 1.47 (s, 9H, rotamer A), 1.41 (s, 9H, rotamer B) ppm; MS (ESI): *m/z* calcd. for $[C_{13}H_{24}N_2O_4]^+$: 273.18 $[M+H]^+$, found: 273.12.

Tert-butyl (2S,4S)-4-(dimethylamino)-2-formylpyrrolidine-1-carboxylate (**11**)

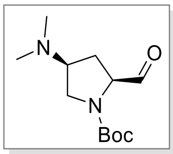

$C_{12}H_{22}N_2O_3$
MW: 242.32 g • mol^{-1}

Ester **10** (114 mg, 420 μmol, 1 equiv.) was dissolved in dry CH_2Cl_2 (3.5 mL, 0.12 M) and treated following General Procedure A. Crude product was purified by column chromatography (gradient: from 2% to 5% of MeOH in CH_2Cl_2). Final aldehyde **11** was obtained as a colourless oil (58 mg, 58%).

R_f = 0.55 (9:1 CH_2Cl_2/MeOH); MS (ESI): *m/z* calcd. for $[C_{12}H_{22}N_2O_3]^+$: 243.17 $[M+H]^+$, found: 243.14.

Tert-butyl (2S,4S)-4-(dimethylamino)-2-((methylamino)methyl) pyrrolidine-1-carboxylate (**12**)

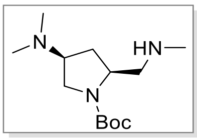

$C_{13}H_{27}N_3O_2$
MW: 257.38 g • mol^{-1}

Aldehyde **11** (58 mg, 240 μmol, 1 equiv.) was dissolved in CH_2Cl_2/AcOH (5 mL + 500 μL) under nitrogen atmosphere and treated with methylamine (33% wt in absolute ethanol, 120 μL, 960 μmol, 4 equiv.) following General Procedure B. The crude product was then purified by HPLC (eluent A: H_2O + 0.1% AcOH; eluent B: MeCN, ramp from 10% B (at min 1) to 38% B (at min 12), t_R (product): 5.6 min). The purified product was lyophilized to give amine **12** as a yellow solid (53 mg, 86% over two steps).

MS (ESI): *m/z* calcd. for $[C_{13}H_{27}N_3O_2]^+$: 258.22 $[M+H]^+$, found: 258.18.

Sp6-CPT

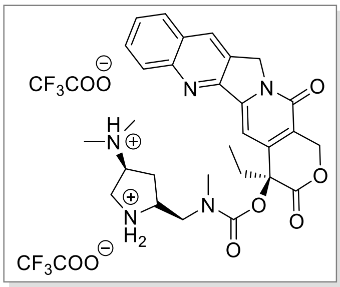

$C_{29}H_{33}N_5O_5$
MW: 531.61 g • mol^{-1} + TFA

To a solution of **CPT-PNP** (6 mg, 13 μmol, 1 equiv.) in dry CH_2Cl_2 was added compound **12** (10 mg, 39 μmol, 3 equiv.) under nitrogen atmosphere followed by DIPEA (9 μL, 52 μmol, 4 equiv.). The reaction was stirred at 20 °C for 2h. The crude product [R_f = 0.40 (9:1 CH_2Cl_2/MeOH)] was dissolved in dry CH_2Cl_2 (800 μL, 0.05 M) and treated following General Procedure C. The crude product was then purified by HPLC (eluent A: H_2O + 0.1% TFA; eluent B: MeCN, ramp from 0% B (at min 1) to 60% B (at min 10.5), t_R (product): 8.9 min). The pure fractions lyophilized to give carbamate **Sp6-CPT** as a yellow solid (21 mg, 96% over two steps).

MS (ESI): *m/z* calcd. for $[C_{29}H_{33}N_5O_5]^+$: 532.26 [*M*+H]$^+$, found: 532.45; HRMS (ESI) *m/z* calcd. for $[C_{29}H_{33}N_5O_5]^+$: 532.2560 [M+H]$^+$, found: 532.2563, *m/z* calcd. for $[C_{29}H_{33}N_5O_5]^+$: 554.2379 [M +Na]$^+$, found: 554.2380.

Synthesis of Sp7-CPT

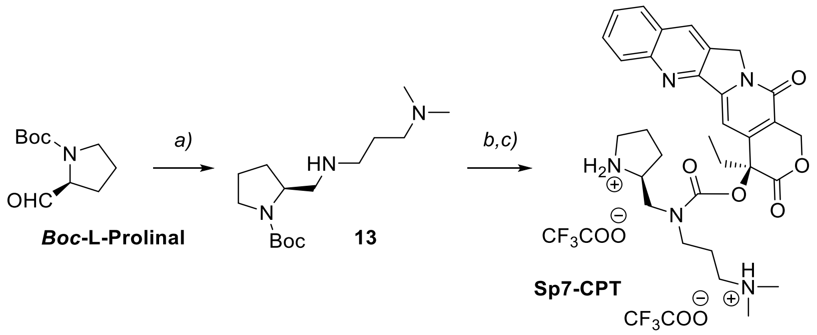

Scheme S5. REAGENTS AND CONDITIONS: *a) N*,*N*-dimethyl-1,3-propanediamine, $NaBH(OAc)_3$, AcOH in dry CH_2Cl_2, r.t., overnight; *b)* **CPT-PNP** in dry CH_2Cl_2, r.t. 3h;. *c)* TFA/ CH_2Cl_2 1:2, 0 °C to r.t., 45 min.

Tert-butyl 2-(((3-(dimethylamino)propyl)amino)methyl)pyrrolidine-1-carboxylate (**13**)

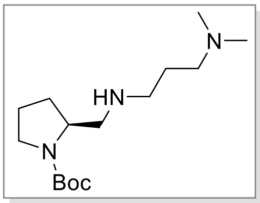

$C_{15}H_{31}N_3O_2$
MW: 285.43 g • mol^{-1}

Boc-(L)-Prolinal (187 µL, 1 mmol, 1 equiv.) was dissolved in a 10% AcOH solution in CH_2Cl_2 (20 mL + 2 mL AcOH) under nitrogen atmosphere and treated following General Procedure B with *N*,*N*-dimethyl-1,3-propanediamine (500 µL, 4 mmol, 4 equiv.). The product was obtained as a colourless oil (260 mg, 91%).

^{1}H NMR (400 MHz, MeOD) *δ* 3.89 (bs, 1H), 3.36 (m, 1H), 2.79 (dd, *J* = 11.7, 4.9 Hz, 1H), 2.67-2.60 (m, 2H), 2.55 (dd, *J* = 7.6, 11.7 Hz, 1H), 2.39-2.34 (m, 2H), 2.25 (s, 6H), 2.02-1.79 (m, 5H), 1.74-1.66 (m, 2H), 1.47 (s, 9H) ppm.

N,N-dimethylpropanediamine-CPT module (**Sp7-CPT**)

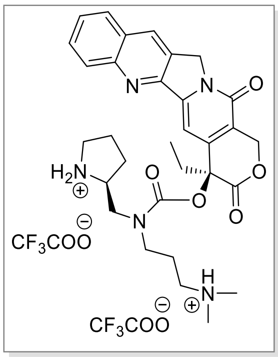

$C_{31}H_{37}N_5O_5$
MW: 559.67 g • mol^{-1} + TFA

To a solution of **CPT-PNP** (7 mg, 14 µmol, 1 equiv.) in dry CH_2Cl_2 was added amine **13** (12 mg, 41 µmol, 3 equiv.) under nitrogen atmosphere followed by DIPEA (10 µL, 56 µmol, 4 equiv.). The reaction was

stirred at 20 °C for 2h. The crude product [R_f = 0.24 (9:1 CH_2Cl_2/MeOH); MS (ESI): *m/z* calcd. for $[C_{36}H_{45}N_5O_7]^+$: 660.34 $[M+H]^+$, found: 660.32; *m/z* calcd. for $[C_{36}H_{45}N_5O_7]^+$: 682.32 $[M+Na]^+$, found: 682.33.] was dissolved in dry CH_2Cl_2 (800 µL, 0.05 M) and treated following General Procedure C. The crude product was then purified by HPLC [eluent A: H_2O + 0.1% TFA; eluent B: MeCN, ramp from 10% B (at min 1) to 60% B (at min 10.5), t_R (product): 8.6 min]. The purified product was lyophilized to give **Sp7-CPT** as a yellow solid (20 mg, 90% over two steps).

MS (ESI): *m/z* calcd. for $[C_{31}H_{37}N_5O_5]^+$: 560.29 $[M+H]^+$, found: 560.41; HRMS (ESI) *m/z* calcd. for $[C_{31}H_{37}N_5O_5]^+$: 560.2873 $[M+H]^+$, found: 560.2870.

Synthesis of Sp8-CPT

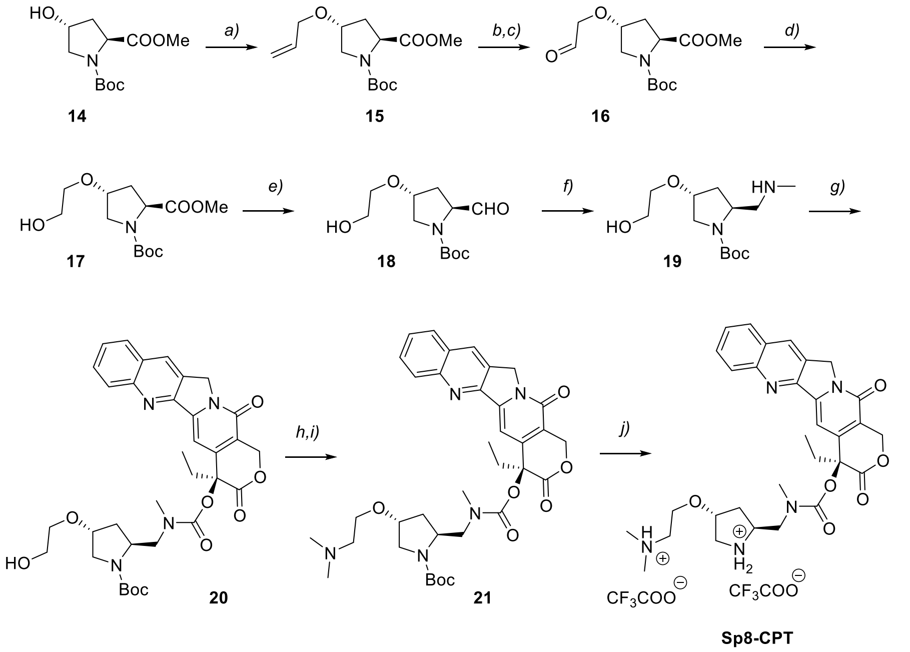

Scheme S6. REAGENTS AND CONDITIONS: *a)* allyl bromide, KHMDS, THF, 0 °C to r.t., overnight; *b*) OsO_4 (2% mol), NMO·H_2O, THF/H_2O (2:1), 0 °C to r.t., overnight; *c*) $NaIO_4$, THF/H_2O (2:1), r.t., 1h; *d*) $NaBH_4$, MeOH, 0 °C to r.t., 2.5h; *e*) DIBAL-H, CH_2Cl_2, -78 °C, 1.5h; *f*) $MeNH_2$, $NaBH(OAc)_3$, CH_2Cl_2, AcOH, r.t., 60h; *g*) **CPT-PNP**, DIPEA, CH_2Cl_2, r.t., 3h; *h*) DMP, CH_2Cl_2, 0 °C to r.t., 1 h; *i*) $MeNH_2$, $NaBH(OAc)_3$, CH_2Cl_2, AcOH, r.t., overnight; *j*) TFA/ CH_2Cl_2 1:2, 0 °C to r.t., 1h.

Boc-Hyp(All)-OMe (**15**)

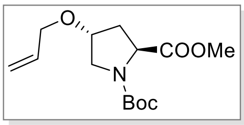

$C_{14}H_{23}NO_5$
MW: 285.34 g • mol^{-1}

To a stirred solution of Boc-Hyp-OMe **14**[3] (491 mg, 2.00 mmol, 1 equiv.) in dry THF (8 mL) cooled to 0 °C under nitrogen atmosphere was added a 0.5 M solution of KHMDS in toluene (4.8 mL, 2.40 mmol, 1.2 equiv.). The mixture was stirred for 5 min at 0 °C, then allyl bromide (208 µL, 2.40 mmol, 1.2 equiv.) was added at 0 °C. The reaction mixture was stirred overnight at r.t. and then water (5 mL) was added

and THF was evaporated in vacuo. The aqueous layer was extracted with CH_2Cl_2 (3 x 30 mL). The combined organic layer was dried, and concentrated. The crude product was purified through flash chromatography (8:2, Hex/AcOEt), to give ether **15** as a colorless oil (374 mg, 65%).

R_f = 0.34 (8:2, Hex/AcOEt stained with ceric ammonium molybdate solution); ^{1}H NMR (400 MHz, $CDCl_3$) δ 5.86 (m, 1H), 5.30-5.14 (m, 2H), 4.37 (m, 1H), 4.10 (m, 1H), 4.02-3.91 (m, 2H), 3.72 (s, 3H, rotamer A), 3.71 (s, 3H, rotamer B), 3.68-3.45 (m, 2H), 2.39-2.03 (m, 2H), 1.47-1.40 (m, 9H) ppm; MS (ESI): *m/z* calcd. for $[C_{14}H_{23}NO_5]^+$: 308.15 $[M+Na]^+$, found: 308.07.

Boc-Hyp(2-oxoethoxy)-OMe (**16**)

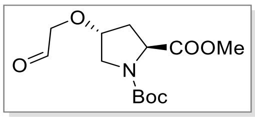

$C_{13}H_{21}NO_6$
MW: 287.31 g • mol^{-1}

To an ice-cold solution of terminal alkene **15** (200 mg, 700 µmol, 1 equiv.) and 4-methylmorpholine *N*-oxide monohydrate (194 mg, 1.44 mmol, 2 equiv.) in a 2:1 THF/H_2O mixture (4.5 mL), OsO_4 (2.5 wt% solution in *t*-BuOH, 176 µL, 14 µmol, 0.02 equiv.) was added. The mixture was stirred for 3 h at 0 °C and then allowed to warm to r.t. and stirred overnight. Solid sodium hydrogen sulfite was added and the mixture as stirred for 1 h at r.t. The mixture was filtered through a pad of silica and rinsed with THF. Volatiles removal led to the crude 1,2-diol intermediate as a yellow oil (288 mg), which was used in the following step without purification. The diol was dissolved in a 2:1 THF/H_2O solution (6.9 mL) and sodium periodate (305 mg, 1.43 mmol, 2 equiv.) was added. The mixture was stirred at r.t. for 1 h. Water (4 mL) was added and THF was evaporated in vacuo. The aqueous layer was extracted with CH_2Cl_2 (3 x 30 mL). The combined organic layers were dried, filtered and concentrated in vacuo, to give aldehyde **16** as a yellow oil (207 mg, quant), which was rapidly used in the following step.

R_f = 0.36 (8:2, AcOEt/Hex stained with ceric ammonium molybdate solution).

Boc-Hyp(2-hydroxyethoxy)-OMe (**17**)

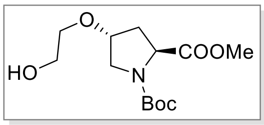

$C_{13}H_{23}NO_6$
MW: 289.33 g • mol^{-1}

Aldehyde **16** (206 mg, 0.72 mmol, 1 equiv.) was dissolved in dry MeOH (7.2 mL) under nitrogen atmosphere and cooled to 0 °C. $NaBH_4$ (41 mg, 1.08 mmol, 1.5 equiv.) was added at 0 °C and then the reaction mixture was allowed to warm to r.t. The reaction mixture was stirred at r.t. for 2.5 h. After concentrating the solvent, 5 mL of a sat. aq. $NaHCO_3$ solution were added and the aqueous layer was

then extracted with CH_2Cl_2 (3 x 30 mL). The combined organic layers were dried (Na_2SO_4), filtered and concentrated in vacuo. The crude product was purified through flash column chromatography (3% of MeOH in CH_2Cl_2), to give alcohol **17** as a colorless oil (176 mg, 85%).

R_f = 0.40 (95:5, CH_2Cl_2/MeOH stained with $KMnO_4$ and conc. H_2SO_4); ^{1}H NMR (400 MHz, $CDCl_3$) δ 4.39 (m, 1H), 4.17-4.06 (m, 2H), 3.74-3.70 (m, 4H), 3.69-3.45 (m, 5H), 2.47-2.03 (m, 2H), 1.47-1.41 (m, 9H) ppm; MS (ESI): *m/z* calcd. for $[C_{13}H_{23}NO_6]^+$: 312.14 $[M+Na]^+$, found: 312.19.

Boc-Hyp(2-hydroxyethoxy)-CHO (**18**)

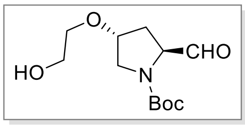

$C_{12}H_{21}NO_5$
MW: 259.30 g • mol^{-1}

A solution of methylester **17** (109 mg, 380 µmol, 1 equiv.) in dry CH_2Cl_2 (3.8 mL) and a solution of DIBAL-H (1 M in toluene, 1 mL) under nitrogen atmosphere were separately cooled to -78 °C in an acetone/dry-ice bath. Portions of the cold DIBAL-H solution (10 x 80 µL, 790 µmol, 2.1 equiv.) were rapidly transferred to the stirring **17** solution. The mixture was then stirred at -78 °C for 1.5 h, followed by addition of CH_2Cl_2 (30 mL) at -78 °C. The mixture was then warmed to 0 °C (ice-water bath), followed by sequential addition of water (32 µL), 15% aq. NaOH (32 µL) and again water (79 µL). The resulting emulsion was warmed to r.t. and stirred for 15 min. After the addition of Na_2SO_4, the mixture was stirred for additional 15 min. The mixture was then filtered and concentrated, to give aldehyde **18** as a yellow oil (86 mg, 88%), which was immediately used in the following step, without purification.

R_f = 0.43 (95:5, CH_2Cl_2/MeOH stained with 2,4-dinitrophenylhydrazine).

Boc-Hyp(2-hydroxyethoxy)-CPT (**20**)

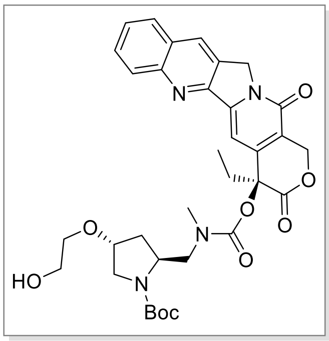

$C_{34}H_{40}N_4O_9$
MW: 648.71 g • mol^{-1}

Aldehyde **18** (86 mg, 330 µmol, 1 equiv.) was dissolved in CH_2Cl_2 (6.5 mL) containing AcOH (28 µL, 1.5 equiv.) under nitrogen atmosphere and treated with methylamine (33% wt in absolute ethanol, 165 µL,

1.33 mmol, 4 equiv.) following General Procedure B. The crude product was purified with flash chromatography (10% MeOH in CH_2Cl_2 + 1% TEA) to obtain secondary amine **19** (49 mg, 200 μmol). The latter was dissolved in dry CH_2Cl_2 (4 mL), followed by addition of **CPT-PNP** (122 mg, 240 μmol, 1.2 equiv.) and DIPEA (138 μL, 800 μmol, 4 equiv.). The mixture was stirred at r.t. for 3 h. After solvent removal, the crude product (200 mg) was purified through flash chromatography (gradient: from 1% to 10% MeOH in CH_2Cl_2), to give carbamate **20** as a yellow solid (107 mg, 84%).

MS (ESI): *m/z* calcd. for $[C_{34}H_{40}N_4O_9]^+$: 671.27 $[M+Na]^+$, found: 671.52.

Boc-Sp8-CPT (**21**)

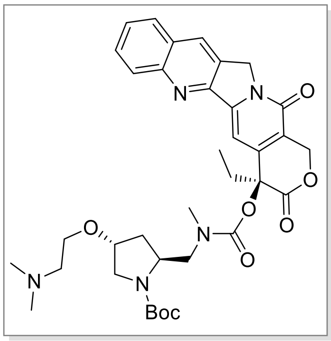

$C_{36}H_{45}N_5O_8$
MW: 675.78 g • mol^{-1}

Alcohol **20** (19 mg, 29 μmol, 1 equiv.) was dissolved in dry CH_2Cl_2 (3 mL) under nitrogen atmosphere and cooled to 0 °C. After the addition of a 0.3 M DMP solution in CH_2Cl_2 (308 μL, 92 μmol, 3 equiv.) at 0 °C, the mixture was warmed to r.t. and stirred for 1 h. After the addition of MeOH (200 μL) at 0 °C and removal of the solvent, the residue was dissolved in a 10% MeOH solution in CH_2Cl_2 and filtered over a pad of silica (rinsed with 10% MeOH solution in CH_2Cl_2). The crude product was dissolved in of CH_2Cl_2 (1 mL) under nitrogen atmosphere and treated with AcOH (4 μL, 1.5 equiv.) and dimethylamine (2 M in THF, 97 μL, 190 μmol, 4 equiv.) following General Procedure B. The crude product was filtered over a pad of silica and eluted with a 9:1 CH_2Cl_2/MeOH mixture (50 mL) and later with a 9:1 CH_2Cl_2/MeOH mixture + 1% TEA (50 mL). After solvent removal, the crude mixture was purified by HPLC (eluent A: H_2O + 0.1% AcOH, eluent B: MeCN, ramp from 15% B at min 0.5 to 70% B at min 9, t_R (product): 6.5 min). The pure fractions were lyophilized to give **21** as a yellow solid (14 mg, 65% over two steps).

MS (ESI): *m/z* calcd. for $[C_{36}H_{46}N_5O_8]^+$: 676.33 $[M+H]^+$, found: 676.39.

Sp8-CPT

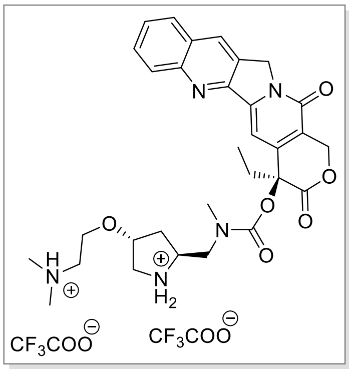

$C_{31}H_{37}N_5O_6$
MW: 575.67 g • mol^{-1} + TFA

21 (5 mg, 7 µmol, 1 equiv.) was dissolved in dry CH_2Cl_2 (250 µL, 0.03 M) and treated with TFA following General Procedure C. The crude product was then purified by HPLC (eluent A: H_2O + 0.1% TFA, eluent B: MeCN, ramp from 15% B at min 1 to 60% B at min 10.5, t_R (product): 7.2 min). The pure fractions were lyophilized to give carbamate **Sp8-CPT** as a yellow solid (4 mg, 71%).

HRMS (ESI): *m/z* calcd. for $[C_{31}H_{38}N_5O_6]^+$: 576.2817 $[M+H]^+$, found: 576.2831.

Synthesis of Sp1-R848, Sp2-R848, Sp3-R848

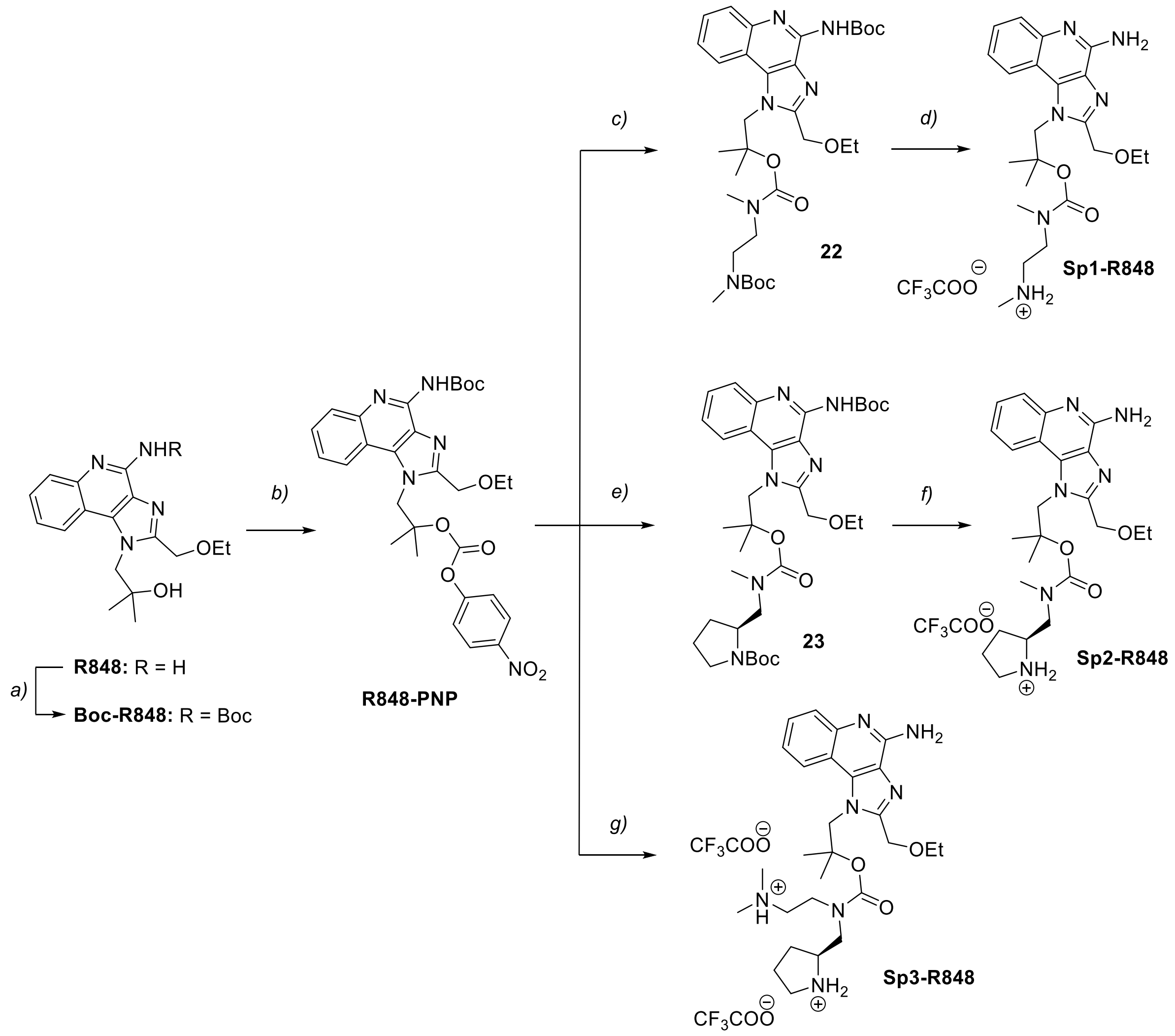

Scheme S7. REAGENTS AND CONDITIONS: *a)* Boc_2O, TEA, THF, 0 °C to r.t. 72 h; *c)* (*N*-Boc)-*N,N'*-dimethylethylenediamine, DIPEA, CH_2Cl_2, r.t. 2.5 h; *d)* TFA/ CH_2Cl_2, r.t. 1 h; *e)* Boc-Pro-NHMe, DIPEA, CH_2Cl_2, r.t. 1h 45'; *f)* TFA/ CH_2Cl_2, r.t. 1 h; *g)* **3**, DIPEA, CH_2Cl_2, r.t. 1h 45'; [2] TFA/ CH_2Cl_2, r.t. 1 h.

Boc-R848

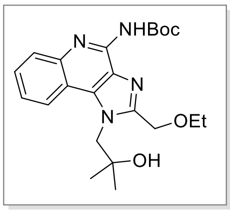

$C_{22}H_{30}N_4O_4$
MW: 414.51 g • mol^{-1}

R848 (Fluorochem, 83 mg, 260 µmol, 1 equiv.) was dissolved in THF (3 ml). Boc_2O (288 mg, 1.32 mmol, 5 equiv.) and TEA (183 µL, 1.32 mmol, 5 equiv.) were added at 0 °C and the reaction was stirred at r.t. for 72 h. The crude material was purified by flash chromatography (eluent 3% MeOH in CH_2Cl_2), to give Boc-R848 as a white foam (95 mg, 88%).

R_f = 0.45 (95:5 CH_2Cl_2/MeOH); ^{1}H NMR (400 MHz, $CDCl_3$) δ 8.19 (d, J = 8.3 Hz, 1H), 8.12 (d, J = 8.2 Hz, 1H), 7.58 (t, J = 7.6 Hz, 1H), 7.45 (t, J = 7.5 Hz, 1H), 4.91 (bs, 2H), 4.78 (bs, 2H), 3.67 (q, J = 7.0 Hz, 2H), 3.20 (s, 1H), 1.59 (s, 9H), 1.32 (bs, 6H), 1.26 (t, J = 7.0 Hz, 3H) ppm; ^{13}C NMR (101 MHz, $CDCl_3$) δ 150.8, 150.6, 144.8, 144.0, 135.4, 129.9, 127.5, 124.4, 119.8, 116.5, 81.4, 71.5, 66.7, 65.0, 56.5, 28.3, 27.9, 14.9 ppm.

R848-PNP

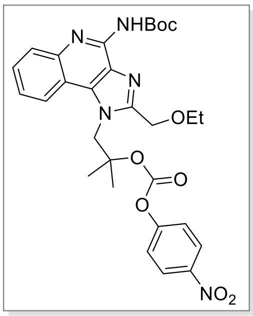

$C_{29}H_{33}N_5O_8$
MW: 579.61 g • mol^{-1}

R848 *N*-Boc (30 mg, 72 µmol, 1 equiv.) was dissolved in CH_2Cl_2 (1 mL) and cooled to 0 °C. DMAP (53 mg, 434 µmol, 6 equiv.) and 4-nitrophenyl chloroformate (44 mg, 217 µmol, 3 equiv.) were added. After 1 h, a white precipitated is observed. The mixture was stirred at r.t. for 4 h. The solution was then diluted with CH_2Cl_2 and concentrated, and the crude product was purified through flash chromatography (eluent 6:4 AcOEt/Hex + 0.1% AcOH) affording carbonate **R848-PNP** (17 mg, 40%, residual 4-nitrophenol was detected by NMR analysis of the collected fractions).

R_f = 0.78 (8:2 AcOEt:Hex 1% AcOH); ^{1}H NMR (400 MHz, CD_2Cl_2) δ 8.40 (d, J = 8.0 Hz, 1H), 8.21 (d, J = 8.3 Hz, 1H), 8.19 (d, J = 9.00 Hz, 2H), 8.10 (d, 4-nitrophenol), 7.78-7.66 (m, 2H), 7.13 (d, J = 9.00 Hz, 2H), 6.92 (d, 4-nitrophenol), 5.00 (bs, 2H), 4.87 (bs, 2H), 3.64 (q, J = 7.00 Hz, 2H), 2.06 (s, AcOH), 1.38

(s, 9H), 1.33 (bs, 6H), 1.22 (t, *J* = 7.00 Hz, 3H) ppm; MS (ESI) *m/z* calcd. for $[C_{29}H_{33}N_5O_8]^+$: 580.24 $[M+H]^+$, found: 580.44; *m/z* calcd. for $[C_{29}H_{33}N_5O_8]^+$: 602.22 $[M+Na]^+$, found: 602.53.

Boc-Sp1-R848 (**22**)

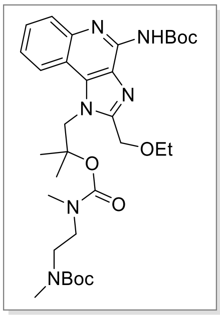

$C_{32}H_{48}N_6O_7$
MW: 628.77 g • mol^{-1}

Carbonate **R848-PNP** (8 mg, 14.3 μmol, 1 equiv.) was dissolved in CH_2Cl_2 (1.0 ml) and (*N*-Boc)-*N,N'*-dimethylethylenediamine[4] (12 mg, 63 μmol, 4.4 equiv.) and DIPEA (5.5 μL, 31.5 μmol, 2.2 equiv.) were added. The reaction was stirred at r.t. for 2.5 h and the crude material was purified by flash chromatography (7:3 AcOEt:Hex + 0.1% AcOH), to give carbamate **22** as a yellow oil (6 mg, 68%).

R_f = 0.27 (7:3 AcOEt:Hex 1% AcOH); MS (ESI) *m/z* calcd. for $[C_{32}H_{48}N_6O_7]^+$: 629.37 $[M+H]^+$, found: 629.15; *m/z* calcd. for $[C_{32}H_{48}N_6O_7]^+$: 651.35 $[M+Na]^+$, found: 651.25.

Sp1-R848

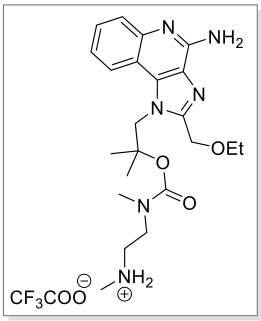

$C_{22}H_{32}N_6O_3$
MW: 428.54 g • mol^{-1} + TFA

Carbamate **22** (6 mg, 9.7 μmol, 1 equiv.) was dissolved in CH_2Cl_2 (324 μL) and treated with TFA following General Procedure C. After solvent removal, the crude material was purified by HPLC [eluent A: H_2O + 0.1% TFA; eluent B: MeCN, ramp from 10% B (at min 1) to 60% B (at min 10), t_R (product): 6.9 min]. The pure product was then lyophilized to give **Sp1-R848** as a colorless solid (3 mg, 83%).

MS (ESI) *m/z* calcd. for $[C_{22}H_{32}N_6O_3]^+$: 429.26 $[M+H]^+$, found: 429.35; HRMS (ESI) *m/z* calcd. for $[C_{22}H_{32}N_6O_3]^+$: 429.2614 $[M+H]^+$, found: 429.2615.

Boc-Sp2-R848 (**23**)

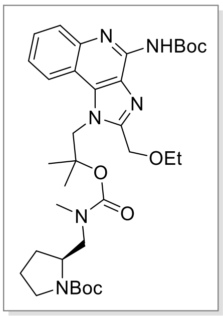

$C_{34}H_{50}N_6O_7$
MW: 654.81 g • mol^{-1}

Carbonate **R848-PNP** (14 mg, 24.2 µmol, 1 equiv.) was dissolved in CH_2Cl_2 (1.7 ml). Secondary amine Boc-Pro-NHMe[4] (23 mg, 105.8 µmol, 4.4 equiv.) and DIPEA (9.3 µL, 96.6 µmol, 2.2 equiv.) were added and the mixture was stirred at r.t. for 1.5 h. Solvent was removed and the crude material was purified by flash chromatography (eluent 1% MeOH in CH_2Cl_2), to give carbamate **23** (19 mg, quant).

R_f = 0.72 (9:1 AcOEt:Hex); MS (ESI) *m/z* calcd. for $[C_{34}H_{50}N_6O_7]^+$: 655.38 [*M*+H]$^+$, found: 655.37; *m/z* calcd. for $[C_{34}H_{50}N_6O_7]^+$: 677.36 [*M*+Na]$^+$, found: 677.39.

Sp2-R848

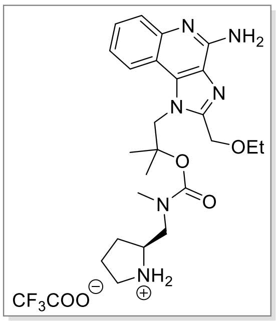

$C_{24}H_{34}N_6O_3$
MW: 454.58 g • mol^{-1} + TFA

Carbamate **23** (16 mg, 24.2 µmol, 1 equiv.) was dissolved in CH_2Cl_2 (783 µL) and treated following General Procedure C. After solvent removal, the crude material was purified by HPLC [eluent A: H_2O + 0.1% TFA; eluent B: MeCN, ramp from 10% B (at min 1) to 60% B (at min 10), t_R (product): 7 min]. The pure product was then lyophilized to give **Sp2-R848** as a colorless solid (18 mg, quant.).

MS (ESI) *m/z* calcd. for $[C_{24}H_{34}N_6O_3]^+$: 455.28 [*M*+H]$^+$, found: 455.29; HRMS (ESI) *m/z* calcd. for $[C_{24}H_{35}N_6O_3]^+$: 455.2771 [*M*+H]$^+$, found: 455.2776.

Sp3-R848

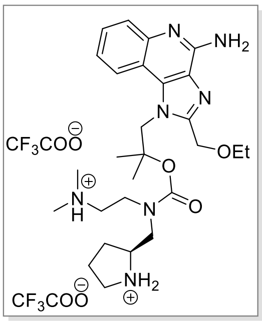

$C_{27}H_{41}N_7O_3$
MW: 511.67 g • mol^{-1} + TFA

Carbonate **R848-PNP** (8 mg, 14 µmol, 1 equiv.) was dissolved in CH_2Cl_2 (1 ml), cooled to 0 °C. Amine **3** (5 mg, 42 µmol, 3 equiv.) and DIPEA (7 µL, 42 µmol, 3 equiv.) were added. The reaction was stirred at rt for 24 h. The crude material was purified by flash chromatography (eluent 5% MeOH in CH_2Cl_2) and the resulting carbamate was dissolved in CH_2Cl_2 (200 µL) and treated with TFA following General Procedure C. After solvent removal, the crude material was purified by HPLC [eluent A: H_2O + 0.1% TFA; eluent B: MeCN, ramp from 10% B (at min 1) to 50% B (at min 9), t_R (product): 8.2 min]. The pure product was then lyophilized to give **Sp3-R848** as a white solid (3 mg, 31%).

HRMS (ESI) *m/z* calcd. for $[C_{27}H_{41}N_7O_3]^+$: 512.3349 $[M+H]^+$, found: 512.3352.

Carbamate Cleavage Studies

Experimental Procedure for Sp-CPT Modules

Stock solutions of lyophilized SI spacer-CPT modules (concentration: 10 mM in DMSO) were diluted 1:10 (final concentration: 1 mM) with 25 mM phosphate buffer (pH 7.4). Immediately after preparation, the mixtures were incubated at 37 °C. Aliquots were collected at different time points and diluted 1:4 (final concentration: 250 μM) with a blocking buffer (8:2 H_2O/CH_3CN + 0.2% TFA).

The diluted aliquots were injected into an analytical HPLC-PDA system (see Materials and Methods), using the following parameters:

Eluent A	H_2O + 0.1% TFA
Eluent B	CH_3CN + 0.1% TFA
Flow Rate	1 mL/min
Gradient	From 10% B to 50% B in 26 min.
UV analysis	254 nm

Areas under the curve (AUC) of the detected peaks were measured using software associated to the HPLC systems. The rate of free OH release from the starting carbamate were obtained by calculating the relative ratios of AUC values corresponding to the amine-bearing prodrug and the free payload. Data were plotted versus time and half-lives ($t_{1/2}$) were calculated by non-linear fitting (exponential, one-phase decay) using GraphPad Prism software.

Experimental Procedure for Sp-R848 modules

Stock solutions of lyophilized SI spacer-R848 modules were diluted with further DMSO and 25 mM phosphate buffer (pH 7.4) according to the following scheme:

	**Sp1-R848**	**Sp2-R848**	**Sp3-R848**
Stock solution concentration in DMSO	20 mM	100 mM	10 mM
Sample (in DMSO) (Volume % Total)	5% - [1 mM] final	1% - [1 mM] final	10% - [1 mM] final
Neat DMSO (Volume % Total)	5%	9%	-
Aq. Buffer (Volume % Total)	90%	90%	90%

Immediately after preparation, the mixtures were incubated at 37 °C and aliquots were collected at different time points and diluted 1:5 (final concentration: 200 μM) with a blocking buffer (8:2 H_2O/CH_3CN + 0.2% TFA).

The diluted aliquots were injected into an analytical HPLC-PDA system, using the following parameters:

Eluent A	H_2O + 0.1% TFA
Eluent B	CH_3CN + 0.1% TFA
Flow Rate	1 mL/min
Gradient	From 5% B to 35% B in 26 min.
UV analysis	254 nm

Areas under the curve (AUC) of the detected peaks were measured using software associated to the HPLC systems. The rate of free OH release from the starting carbamate were obtained by calculating the relative ratios of AUC values corresponding to the amine-bearing prodrug and the free payload. Data were plotted versus time and half-lives ($t_{1/2}$) were calculated by non-linear fitting (exponential, one-phase decay) using GraphPad Prism software.

Computational Studies

All calculations were run using the Schroödinger suite of programs through the Maestro graphical interface.[6] The following steps were implemented to identify relevant minimum-energy conformations of the model carbamates in their main ionization state at pH 7.5 (i.e. protonated pyrrolidine in **Sp2-tBu**, protonated pyrrolidine and tertiary amine in **Sp3-tBu**).

Molecular Mechanics calculations. Monte Carlo/energy minimization (MC/EM) conformational searches[7] of carbamates **Sp2-tBu** and **Sp3-tBu** were performed within the framework of MacroModel version 11.1,[8] using the OPLS3 force field[9] and the implicit water GB/SA solvation model,[10] to generate starting geometries for subsequent DFT calculations. The exocyclic dihedral angles of each compound were randomly varied with the usage-directed Monte Carlo conformational search. For each search, at least 1000 starting structures for each variable torsion angle were generated and minimized until the gradient was <0.05 $kJÅ^{-1}mol^{-1}$ using the truncated Newton–Raphson algorithm.[11] Duplicate conformations and those with energy >5 $kcalmol^{-1}$ above the global minimum were discarded.

DFT calculations. Representative minimum-energy geometries obtained from the MC/EM conformational search were fully optimized with DFT calculations at the B3LYP/6-31G* level of theory using the Jaguar version 9.1.[12] Default convergence criteria were employed. Calculations of vibrational frequencies were carried out to ensure that stationary points were true minima on the potential energy surface. Solution phase energies of the obtained stationary points were computed at the same level of theory by single-point energy calculations including the water PBF solvent model (Poisson-Boltzmann Solvation Model in Jaguar).[12] Representative DFT minimum energy structures displaying relative solution energy differences within 3 kcal/mol of the global minimum are shown in Figure S2 (the *syn 2* conformation of **Sp3-tBu** found at 4.13 kcal/mol is included for completeness). The values of the dihedral angles that distinguish these structures are reported in Table S1. pK_a values of the protonated species were calculated on the lowest energy *anti* and *syn* structure pairs by the Jaguar pK_a prediction module.

---

6 Maestro, release 2016-1, Schrödinger, LLC, New York, NY, 2016.

7 G. Chang, W. C. Guida, W. C. Still, *J. Am. Chem. Soc.* 1989, **111**, 4379.

8 MacroModel, version 11.1, Schrödinger, LLC, New York, NY, 2016.

9 K. Roos, C. Wu, W. Damm, M. Reboul, J.M. Stevenson, C. Lu, M.K. Dahlgren, S. Mondal, W. Chen, L. Wang, R. Abel, R.A. Friesner, E.D. Harder, *J. Chem. Theory Comput.* 2019, **15**, 1863.

10 W. C. Still, A. Tempczyk, R. C. Hawley, T. Hendrickson, *J. Am. Chem. Soc.* 1990, **112**, 6127.

11 J. W. Ponder, F. M. Richards, *J. Comput. Chem.* 1987, **8**, 1016.

12 Jaguar, version 9.1, release 14, Schrödinger, LLC, New York, NY, 2016.

Table S1. Dihedral angles of the pyrrolidine-carbamate chain in representative DFT-optimized conformations of **Sp2/3-tBu**. Relative energy differences are calculated from the corresponding solution phase energies (spE).

Compound	Conformation	ΔspE (kcal/mol)	Dihedral angle C-N-C_{carb}-Osp^3 (degrees) 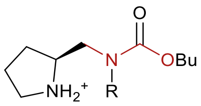	Dihedral angle C-C-N-C_{carb} (degrees) 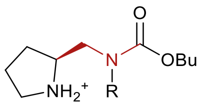	Dihedral angle N_{pyr}-C-C-N_{carb} (degrees) 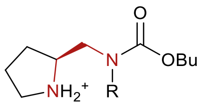
**Sp2-tBu**	*anti* (H-bond)	0.00	-161.1°	-87.1°	67.9
	anti 2 (H-bond)	0.59	-161.0°	83.4°	-72.5°
	syn (H-bond)	2.74	19.0°	-97.9°	61.8°
	anti 3 (H-bond, different ring puckering)	2.88	-166.3°	-68.4°	87.1°
**Sp3-tBu**	*syn* (2 H-bonds)	0.00	15.7°	-94.0°	68.0°
	anti (2 H-bonds)	1.16	-164.8°	-83.6°	72.5°
	anti 2 (1 H-bond)	1.96	-171.5°	-79.3°	73.8°
	anti 3 (1 H-bond)	2.57	171.3°	73.1°	-80.8°
	syn 2 (1 H-bond)	4.13	9.6°	-89.7°	68.5°

Appendix

HPLC Data – Sp-CPT Modules

- **Sp2-CPT** 1 mM
- 25 mM phosphate buffer + 10 % DMSO
- pH 7.4, T: 37 °C

t = 0 h

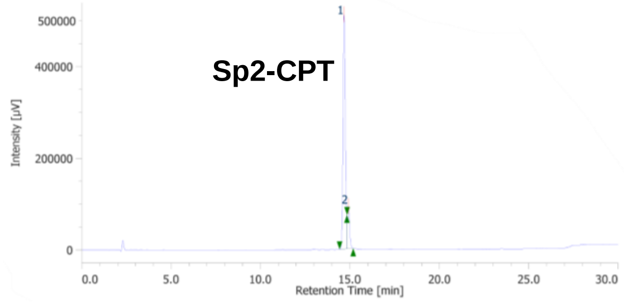

#	Peak Name	CH	tR [min]	Area [µV·sec]	Height [µV]	Area%	Height%	Resolution	Symmetry Factor	Factor
1	Unknown	9	14.7	4722012	511211	85.3	84.694	N/A	N/A	1.00000
2	Unknown	9	14.9	810993	92387	14.7	15.306	N/A	N/A	1.00000
Total				5533005	603598					

t = 1 h

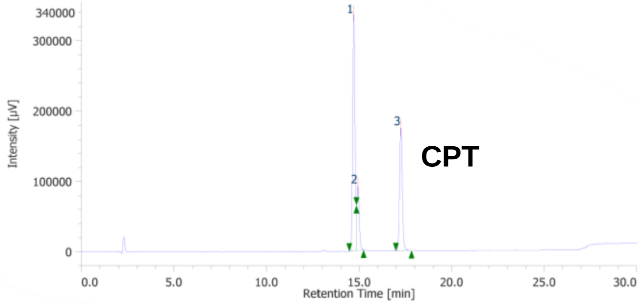

#	Peak Name	CH	tR [min]	Area [µV·sec]	Height [µV]	Area%	Height%	Resolution	Symmetry Factor	Factor
1	Unknown	9	14.7	3086164	337468	53.9	55.859	N/A	N/A	1.00000
2	Unknown	9	14.9	833652	91713	14.6	15.181	N/A	N/A	1.00000
3	Unknown	9	17.3	1804656	174966	31.5	28.961	N/A	1.047	1.00000
Total				5724472	604147					

t = 5 h

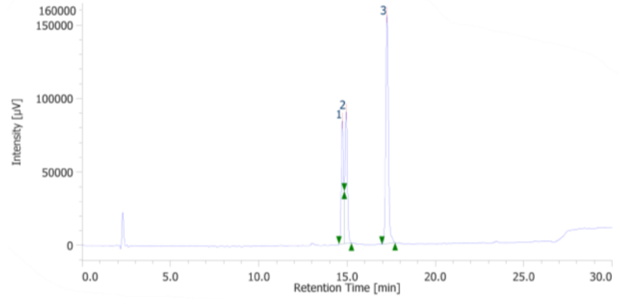

#	Peak Name	CH	tR [min]	Area [µV·sec]	Height [µV]	Area%	Height%	Resolution	Symmetry Factor	Factor
1	Unknown	9	14.7	757222	83696	23.5	25.441	0.843	N/A	1.00000
2	Unknown	9	14.9	841424	89632	26.2	27.245	8.824	N/A	1.00000
3	Unknown	9	17.3	1618228	155658	50.3	47.314	N/A	1.070	1.00000
Total				3216874	328986					

t = 8 h

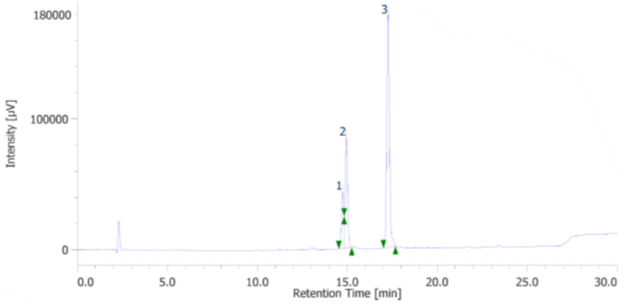

#	Peak Name	CH	tR [min]	Area [µV·sec]	Height [µV]	Area%	Height%	Resolution	Symmetry Factor	Factor
1	Unknown	9	14.7	371878	43211	12.2	14.122	N/A	N/A	1.00000
2	Unknown	9	14.9	805129	84112	26.5	27.488	8.969	N/A	1.00000
3	Unknown	9	17.3	1860656	178669	61.3	58.390	N/A	1.073	1.00000
Total				3037663	305992					

- **Sp3-CPT** 1 mM
- 25 mM phosphate buffer + 10 % DMSO
- pH 7.4, T: 37 °C

t = 0 h

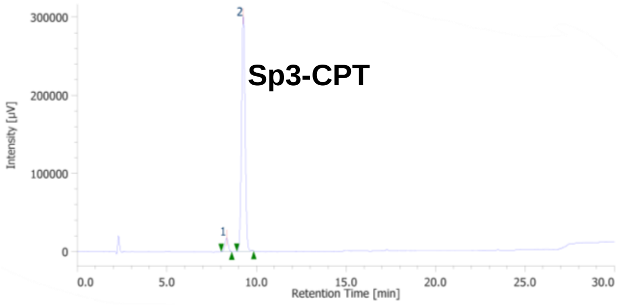

#	Peak Name	CH	tR [min]	Area [µV·sec]	Height [µV]	Area%	Height%	Resolution	Symmetry Factor	Factor
1	Unknown	9	8.34	223487	17741	5.10	5.568	2.674	0.958	1.00000
2	Unknown	9	9.27	4162761	300896	94.9	94.432	N/A	1.022	1.00000
Total				4386248	318637					

t = 1 h

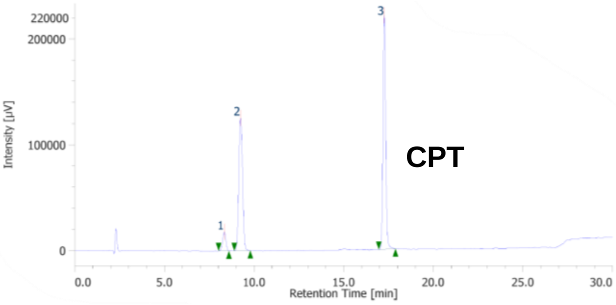

#	Peak Name	CH	tR [min]	Area [µV·sec]	Height [µV]	Area%	Height%	Resolution	Symmetry Factor	Factor
1	Unknown	9	8.33	226422	17859	5.19	4.902	2.485	0.940	1.00000
2	Unknown	9	9.24	1849579	125355	42.4	34.410	24.319	1.058	1.00000
3	Unknown	9	17.3	2285636	221085	52.4	60.688	N/A	1.045	1.00000
Total				4361637	364299					

t = 5 h

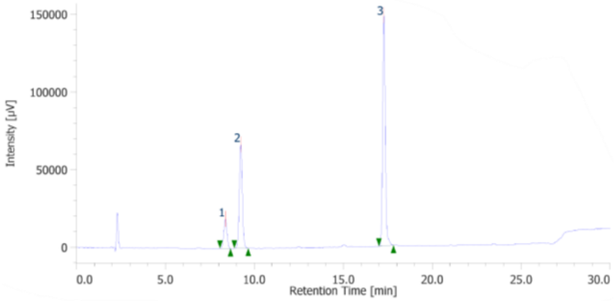

#	Peak Name	CH	tR [min]	Area [µV·sec]	Height [µV]	Area%	Height%	Resolution	Symmetry Factor	Factor
1	Unknown	9	8.37	239732	18940	9.26	8.089	2.697	0.973	1.00000
2	Unknown	9	9.24	799346	66791	30.9	28.524	27.986	1.042	1.00000
3	Unknown	9	17.3	1549516	148425	59.9	63.387	N/A	1.063	1.00000
Total				2588594	234156					

t = 8 h

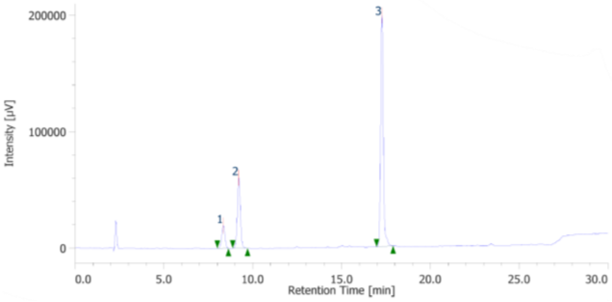

#	Peak Name	CH	tR [min]	Area [µV·sec]	Height [µV]	Area%	Height%	Resolution	Symmetry Factor	Factor
1	Unknown	9	8.35	248693	19530	8.11	7.016	2.704	0.970	1.00000
2	Unknown	9	9.22	723775	60470	23.6	21.725	28.040	1.040	1.00000
3	Unknown	9	17.3	2094513	198343	68.3	71.259	N/A	1.090	1.00000
Total				3066981	278343					

- **Sp4-CPT** 1 mM
- 25 mM phosphate buffer + 10 % DMSO
- pH 7.4, T: 37 °C

t = 0 h

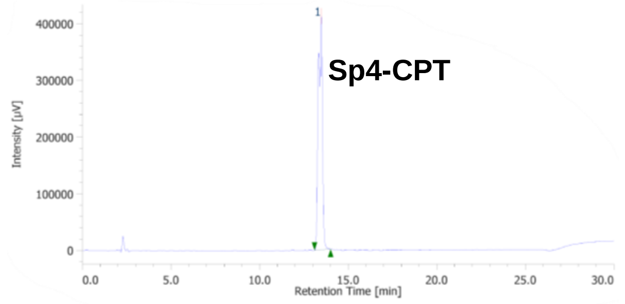

#	Peak Name	CH	tR [min]	Area [μV·sec]	Height [μV]	Area%	Height%	Resolution	Symmetry Factor	Factor
1	Unknown	9	13.5	6716274	411843	100	100.000	N/A	0.802	1.00000
Total				6716274	411843					

t = 1 h

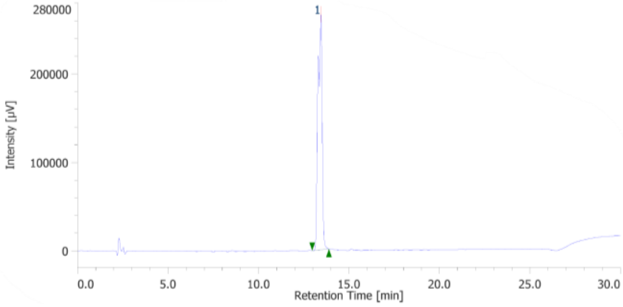

#	Peak Name	CH	tR [min]	Area [μV·sec]	Height [μV]	Area%	Height%	Resolution	Symmetry Factor	Factor
1	Unknown	9	13.4	4242007	265898	100	100.000	N/A	0.801	1.00000
Total				4242007	265898					

t = 5 h

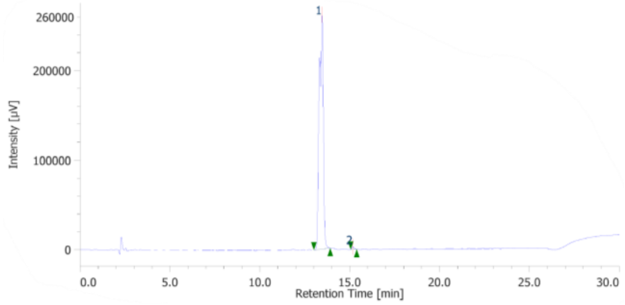

#	Peak Name	CH	tR [min]	Area [μV·sec]	Height [μV]	Area%	Height%	Resolution	Symmetry Factor	Factor
1	Unknown	9	13.5	4125245	261645	99.4	98.935	4.777	0.792	1.00000
2	Unknown	9	15.2	25365	2818	0.611	1.065	N/A	1.288	1.00000
Total				4150610	264463					

t = 8 h

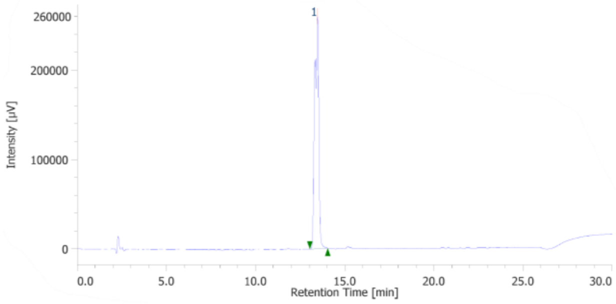

#	Peak Name	CH	tR [min]	Area [μV·sec]	Height [μV]	Area%	Height%	Resolution	Symmetry Factor	Factor
1	Unknown	9	13.5	4113619	259730	100	100.000	N/A	0.800	1.00000
Total				4113619	259730					

- **Sp5-CPT** 1 mM
- 25 mM phosphate buffer + 10 % DMSO
- pH 7.4, T: 37 °C

t = 0 h

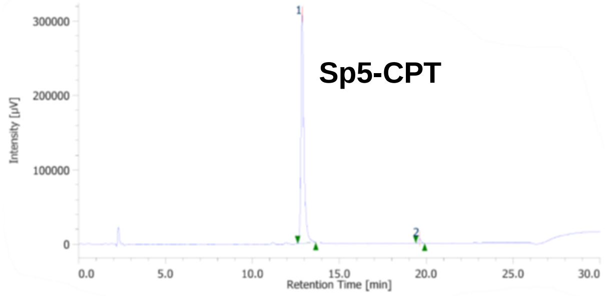

#	Peak Name	CH	tR [min]	Area [μV·sec]	Height [μV]	Area%	Height%	Resolution	Symmetry Factor	Factor
1	Unknown	9	12.9	3646248	307872	98.3	98.042	25.124	1.530	1.00000
2	Unknown	9	19.6	61235	6147	1.65	1.958	N/A	1.147	1.00000
Total				3707483	314019					

t = 1 h

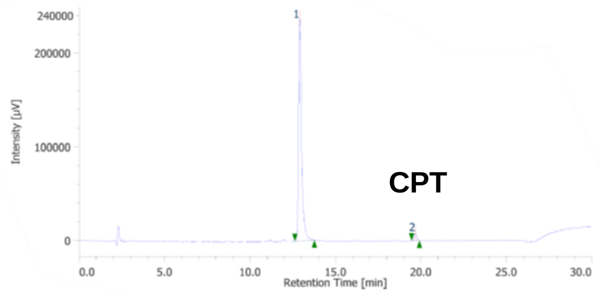

#	Peak Name	CH	tR [min]	Area [μV·sec]	Height [μV]	Area%	Height%	Resolution	Symmetry Factor	Factor
1	Unknown	9	12.9	2835816	237522	97.4	96.923	24.943	1.512	1.00000
2	Unknown	9	19.7	75217	7540	2.58	3.077	N/A	1.005	1.00000
Total				2911033	245062					

t = 5 h

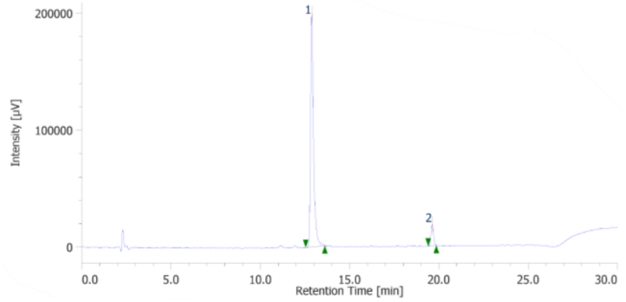

#	Peak Name	CH	tR [min]	Area [μV·sec]	Height [μV]	Area%	Height%	Resolution	Symmetry Factor	Factor
1	Unknown	9	12.9	2309386	199223	92.8	91.633	25.326	1.501	1.00000
2	Unknown	9	19.6	178916	18192	7.19	8.367	N/A	1.014	1.00000
Total				2488302	217415					

t = 8 h

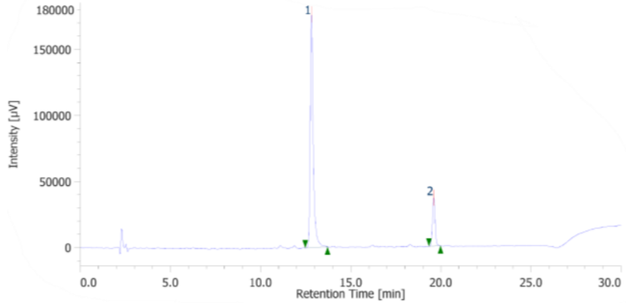

#	Peak Name	CH	tR [min]	Area [μV·sec]	Height [μV]	Area%	Height%	Resolution	Symmetry Factor	Factor
1	Unknown	9	12.8	2060639	176456	84.5	82.848	25.030	1.484	1.00000
2	Unknown	9	19.6	378397	36532	15.5	17.152	N/A	1.065	1.00000
Total				2439036	212988					

- **Sp6-CPT** 1 mM
- 25 mM phosphate buffer + 10 % DMSO
- pH 7.4, T: 37 °C

t = 0 h

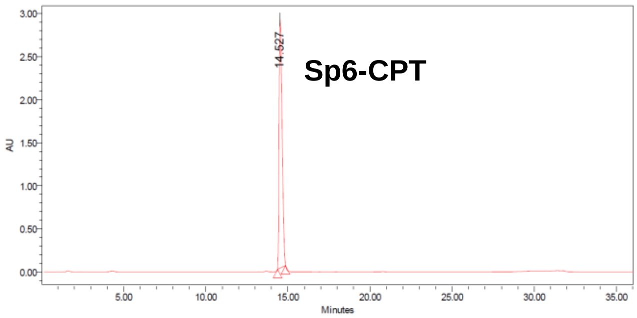

Peak Results

	Name	RT	Area	Height	Amount	Units	% Area
1		14.527	36643380	2907247			100.00

t = 1 h

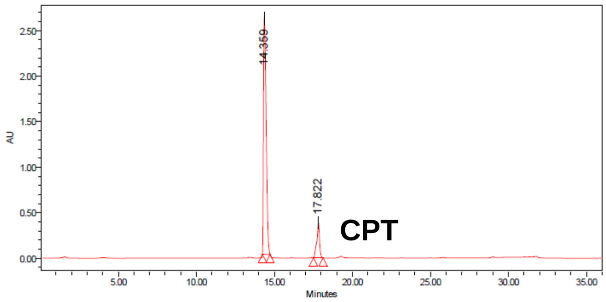

Peak Results

	Name	RT	Area	Height	Amount	Units	% Area
1		14.359	31042815	2612304			86.82
2		17.822	4711409	385275			13.18

t = 5 h

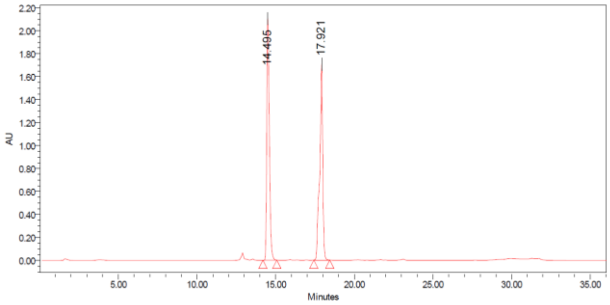

Peak Results

	Name	RT	Area	Height	Amount	Units	% Area
1		14.495	24233891	2124688			51.38
2		17.921	22928771	1702838			48.62

t = 8 h

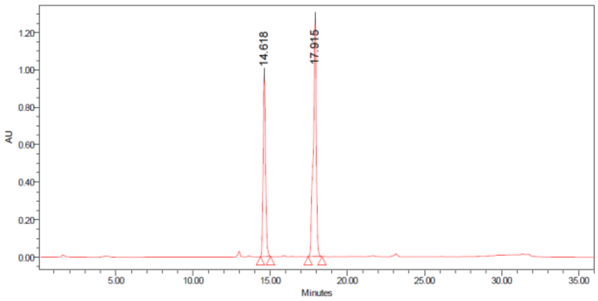

Peak Results

	Name	RT	Area	Height	Amount	Units	% Area
1		14.618	10477199	973577			38.39
2		17.915	16816806	1278753			61.61

- **Sp7-CPT** 1 mM
- 25 mM phosphate buffer + 10 % DMSO
- pH 7.4, T: 37 °C

t = 0 h

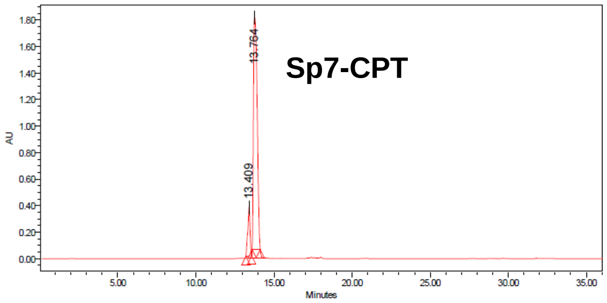

Peak Results

	Name	RT	Area	Height	Amount	Units	% Area
1		13.409	3622851	369571			11.02
2		13.764	29252639	1762699			88.98

t = 1 h

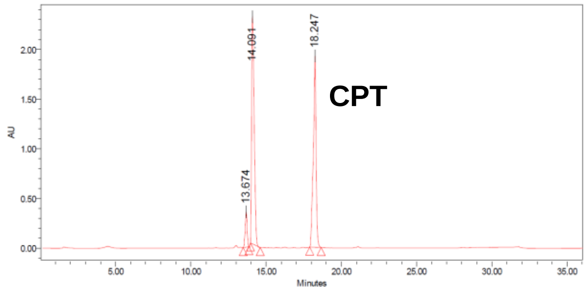

Peak Results

	Name	RT	Area	Height	Amount	Units	% Area
1		13.674	3143655	356143			5.70
2		14.091	27238483	2297449			49.43
3		18.247	24727556	1934821			44.87

t = 5 h

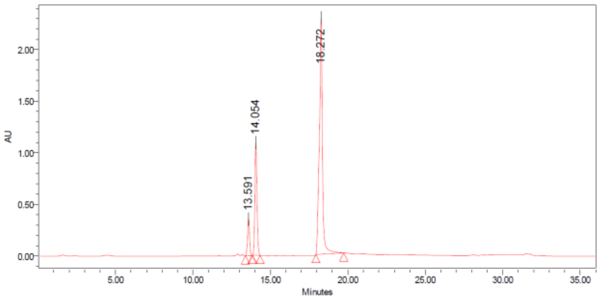

Peak Results

	Name	RT	Area	Height	Amount	Units	% Area
1		13.591	3148905	357395			6.97
2		14.054	10165231	1094594			22.51
3		18.272	31854199	2297143			70.52

t = 8 h

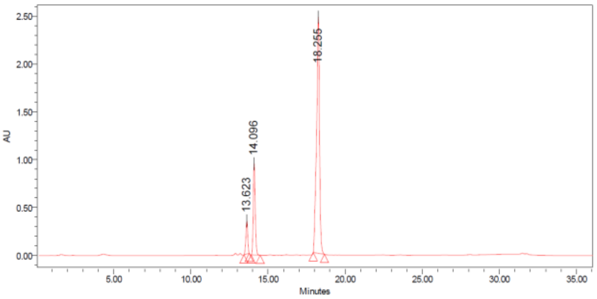

Peak Results

	Name	RT	Area	Height	Amount	Units	% Area
1		13.623	2864549	347109			6.52
2		14.096	8402196	962047			19.13
3		18.255	32658611	2465378			74.35

- **Sp8-CPT** 1 mM
- 25 mM phosphate buffer + 10 % DMSO
- pH 7.4, T: 37 °C

t = 0 h

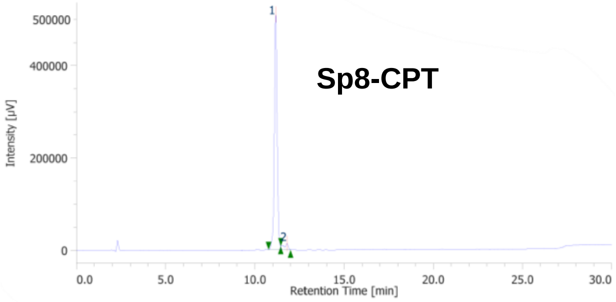

#	Peak Name	CH	tR [min]	Area [µV·sec]	Height [µV]	Area%	Height%	Resolution	Symmetry Factor	Factor
1	Unknown	9	11.2	5536067	509619	95.7	97.418	N/A	0.994	1.00000
2	Unknown	9	11.8	246609	13504	4.26	2.582	N/A	N/A	1.00000
Total				5782676	523123					

t = 1 h

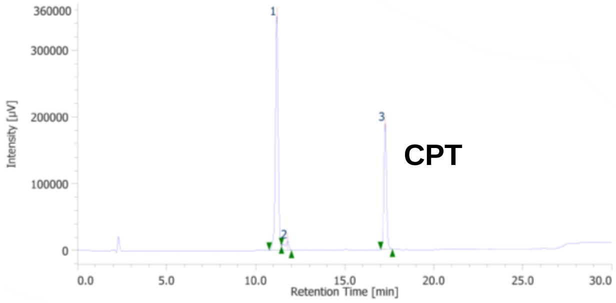

#	Peak Name	CH	tR [min]	Area [µV·sec]	Height [µV]	Area%	Height%	Resolution	Symmetry Factor	Factor
1	Unknown	9	11.2	3755788	351414	63.3	63.417	1.288	0.952	1.00000
2	Unknown	9	11.8	248321	13816	4.19	2.493	11.460	N/A	1.00000
3	Unknown	9	17.3	1925969	188900	32.5	34.089	N/A	1.040	1.00000
Total				5930078	554130					

t = 5 h

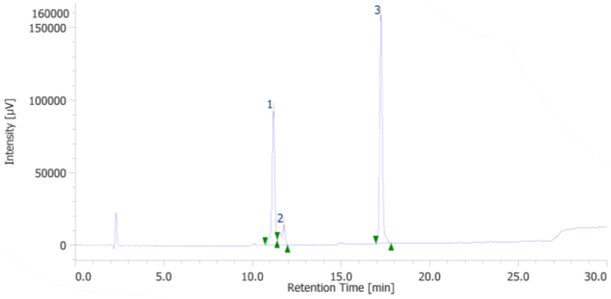

#	Peak Name	CH	tR [min]	Area [µV·sec]	Height [µV]	Area%	Height%	Resolution	Symmetry Factor	Factor
1	Unknown	9	11.2	984771	92661	34.2	35.059	1.330	0.896	1.00000
2	Unknown	9	11.8	237584	13794	8.25	5.219	12.346	N/A	1.00000
3	Unknown	9	17.2	1656835	157849	57.5	59.723	N/A	1.066	1.00000
Total				2879190	264304					

t = 8 h

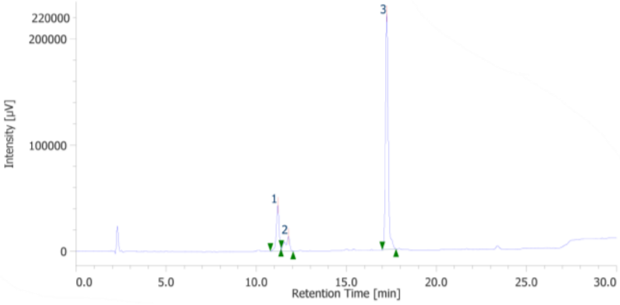

#	Peak Name	CH	tR [min]	Area [µV·sec]	Height [µV]	Area%	Height%	Resolution	Symmetry Factor	Factor
1	Unknown	9	11.2	458103	43196	15.1	15.487	1.337	N/A	1.00000
2	Unknown	9	11.8	243042	13896	7.99	4.982	12.338	N/A	1.00000
3	Unknown	9	17.2	2339308	221827	76.9	79.531	N/A	1.087	1.00000
Total				3040453	278919					

HPLC Data – Sp-R848 Modules

- **Sp1-R848** 1 mM
- 25 mM phosphate buffer + 10 % DMSO
- pH 7.4, T: 37 °C

t = 0 h

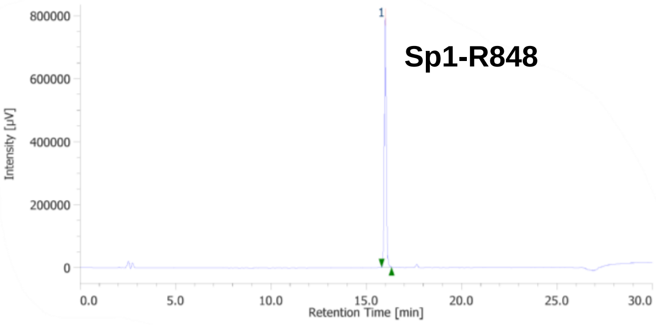

#	Peak Name	CH	tR [min]	Area [µV·sec]	Height [µV]	Area%	Height%	Resolution	Symmetry Factor	Factor
1	Unknown	9	16.0	5678609	793809	100	100.000	N/A	1.092	1.00000
Total				5678609	793809					

t = 2 h

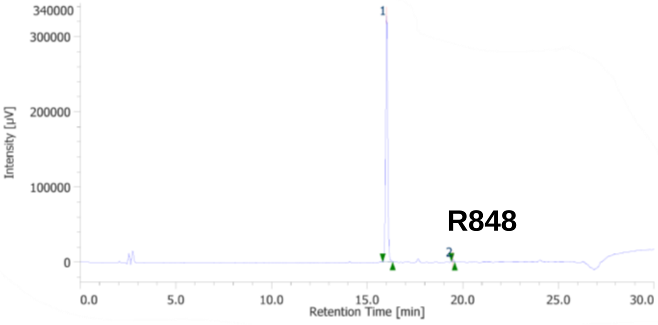

#	Peak Name	CH	tR [min]	Area [µV·sec]	Height [µV]	Area%	Height%	Resolution	Symmetry Factor	Factor
1	Unknown	9	16.0	2425795	327507	99.2	99.053	18.915	1.054	1.00000
2	Unknown	9	19.5	19284	3132	0.789	0.947	N/A	1.018	1.00000
Total				2445079	330639					

t = 5 h

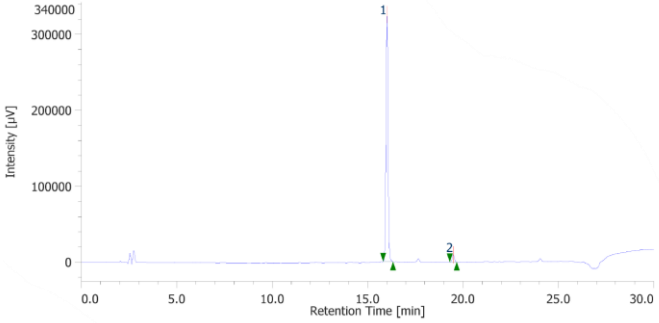

#	Peak Name	CH	tR [min]	Area [µV·sec]	Height [µV]	Area%	Height%	Resolution	Symmetry Factor	Factor
1	Unknown	9	16.0	2419514	325259	97.0	97.153	17.351	1.035	1.00000
2	Unknown	9	19.5	75020	9531	3.01	2.847	N/A	1.021	1.00000
Total				2494534	334790					

t = 8 h

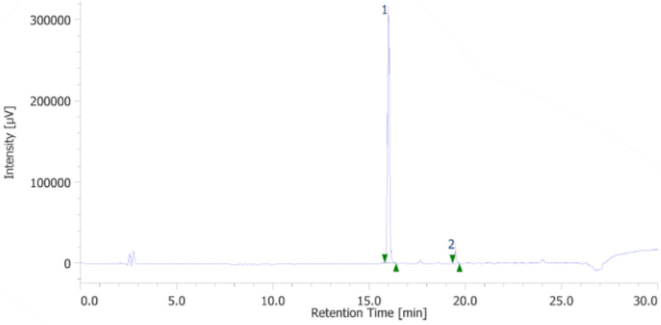

#	Peak Name	CH	tR [min]	Area [µV·sec]	Height [µV]	Area%	Height%	Resolution	Symmetry Factor	Factor
1	Unknown	9	16.0	2272848	306164	95.2	95.433	17.421	1.038	1.00000
2	Unknown	9	19.5	115411	14653	4.83	4.567	N/A	1.080	1.00000
Total				2388259	320817					

- **Sp2-R848** 1 mM
- 25 mM phosphate buffer + 10 % DMSO
- pH 7.4, T: 37 °C

t = 0 h

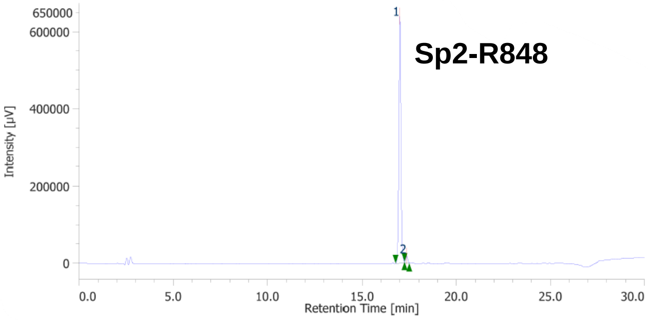

#	Peak Name	CH	tR [min]	Area [μV·sec]	Height [μV]	Area%	Height%	Resolution	Symmetry Factor	Factor
1	Unknown	9	17.0	4942535	639918	97.3	97.265	1.843	1.034	1.00000
2	Unknown	9	17.4	136929	17992	2.70	2.735	N/A	N/A	1.00000
Total				5079464	657910					

t = 2 h

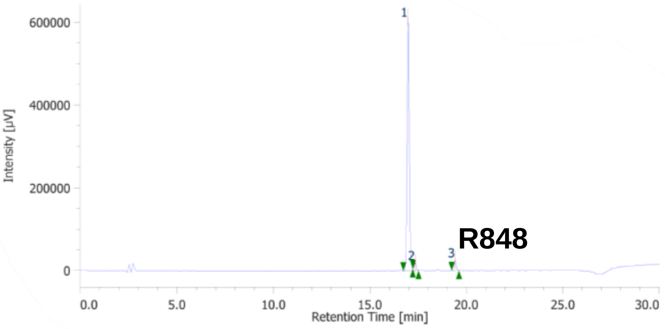

#	Peak Name	CH	tR [min]	Area [μV·sec]	Height [μV]	Area%	Height%	Resolution	Symmetry Factor	Factor
1	Unknown	9	17.0	4712360	612429	93.1	93.355	1.850	1.050	1.00000
2	Unknown	9	17.3	147963	18537	2.92	2.826	9.846	N/A	1.00000
3	Unknown	9	19.4	201432	25057	3.98	3.819	N/A	1.013	1.00000
Total				5061755	656023					

t = 5 h

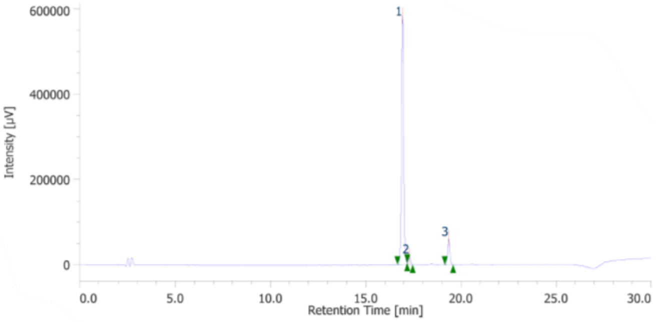

#	Peak Name	CH	tR [min]	Area [μV·sec]	Height [μV]	Area%	Height%	Resolution	Symmetry Factor	Factor
1	Unknown	9	16.9	4580853	583412	87.6	87.849	1.818	1.008	1.00000
2	Unknown	9	17.3	151319	19358	2.89	2.915	9.907	N/A	1.00000
3	Unknown	9	19.4	499298	61340	9.54	9.236	N/A	1.021	1.00000
Total				5231470	664110					

t= 8 h

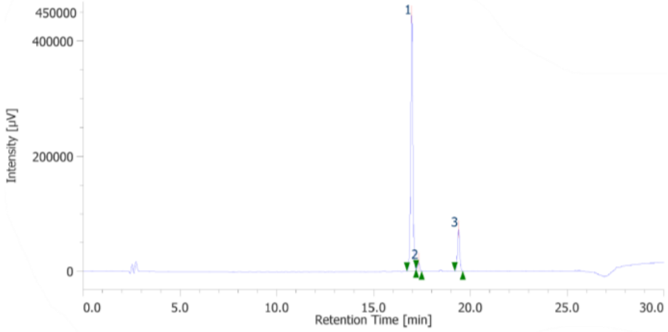

#	Peak Name	CH	tR [min]	Area [μV·sec]	Height [μV]	Area%	Height%	Resolution	Symmetry Factor	Factor
1	Unknown	9	17.0	3489120	446139	83.1	83.426	1.758	0.968	1.00000
2	Unknown	9	17.3	126154	16079	3.01	3.007	10.086	N/A	1.00000
3	Unknown	9	19.4	581844	72551	13.9	13.567	N/A	1.011	1.00000
Total				4197118	534769					

- **Sp3-R848** 1 mм
- 25 mм phosphate buffer + 10 % DMSO
- pH 7.4, T: 37 °C

t = 0 h

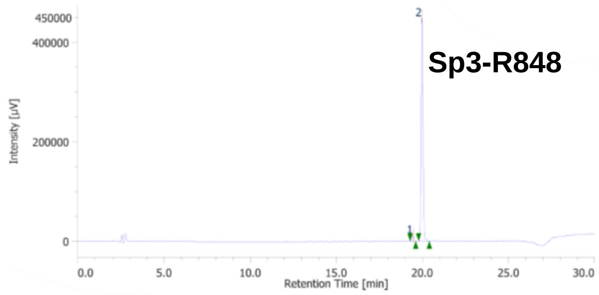

#	Peak Name	CH	tR [min]	Area [µV·sec]	Height [µV]	Area%	Height%	Resolution	Symmetry Factor	Factor
1	Unknown	9	19.5	84535	10698	2.45	2.318	2.567	0.970	1.00000
2	Unknown	9	20.0	3368955	450908	97.6	97.682	N/A	1.097	1.00000
Total				3453490	461606					

t = 2 h

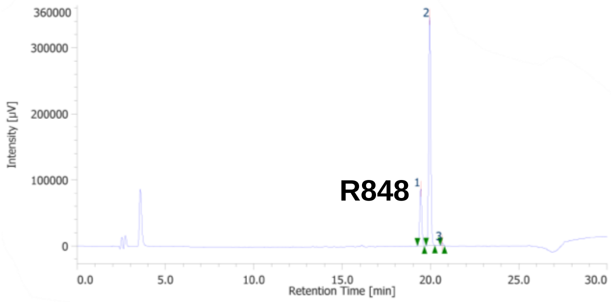

#	Peak Name	CH	tR [min]	Area [µV·sec]	Height [µV]	Area%	Height%	Resolution	Symmetry Factor	Factor
1	Unknown	9	19.4	692868	86311	20.4	19.777	2.483	1.032	1.00000
2	Unknown	9	19.9	2668512	345701	78.7	79.211	3.689	1.075	1.00000
3	Unknown	9	20.7	30567	4420	0.901	1.013	N/A	1.006	1.00000
Total				3391947	436432					

t = 5 h

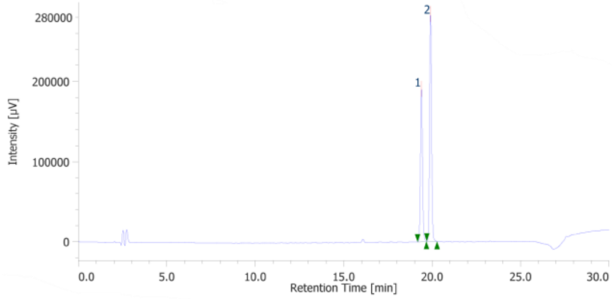

#	Peak Name	CH	tR [min]	Area [µV·sec]	Height [µV]	Area%	Height%	Resolution	Symmetry Factor	Factor
1	Unknown	9	19.4	1522958	190504	41.1	40.208	2.558	1.059	1.00000
2	Unknown	9	19.9	2179195	283292	58.9	59.792	N/A	1.062	1.00000
Total				3702153	473796					

t = 8 h

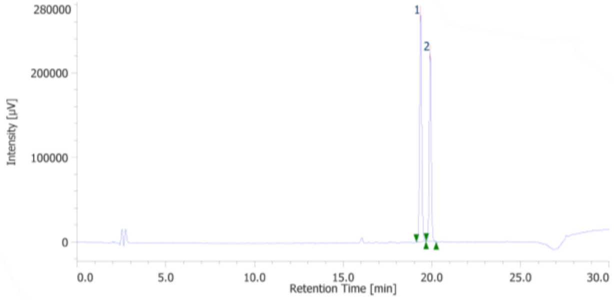

#	Peak Name	CH	tR [min]	Area [µV·sec]	Height [µV]	Area%	Height%	Resolution	Symmetry Factor	Factor
1	Unknown	9	19.4	2165730	268721	56.1	54.701	2.670	1.052	1.00000
2	Unknown	9	19.9	1697404	222531	43.9	45.299	N/A	1.061	1.00000
Total				3863134	491252					

NMR Spectra

Tert-butyl (S)-2-(((2-(dimethylamino)ethyl)amino)methyl)pyrrolidine-1-carboxylate

(**3**)

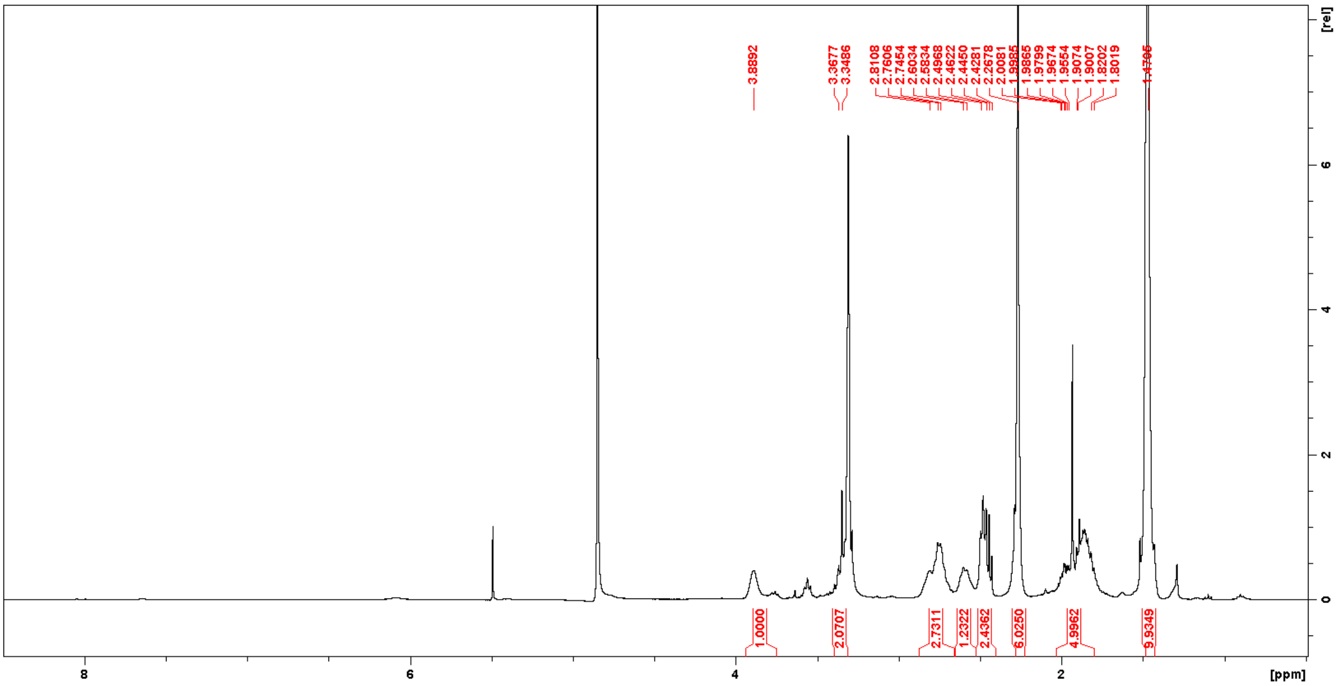

Tert-butyl 2-((methylamino)methyl)piperidine-1-carboxylate (**5**)

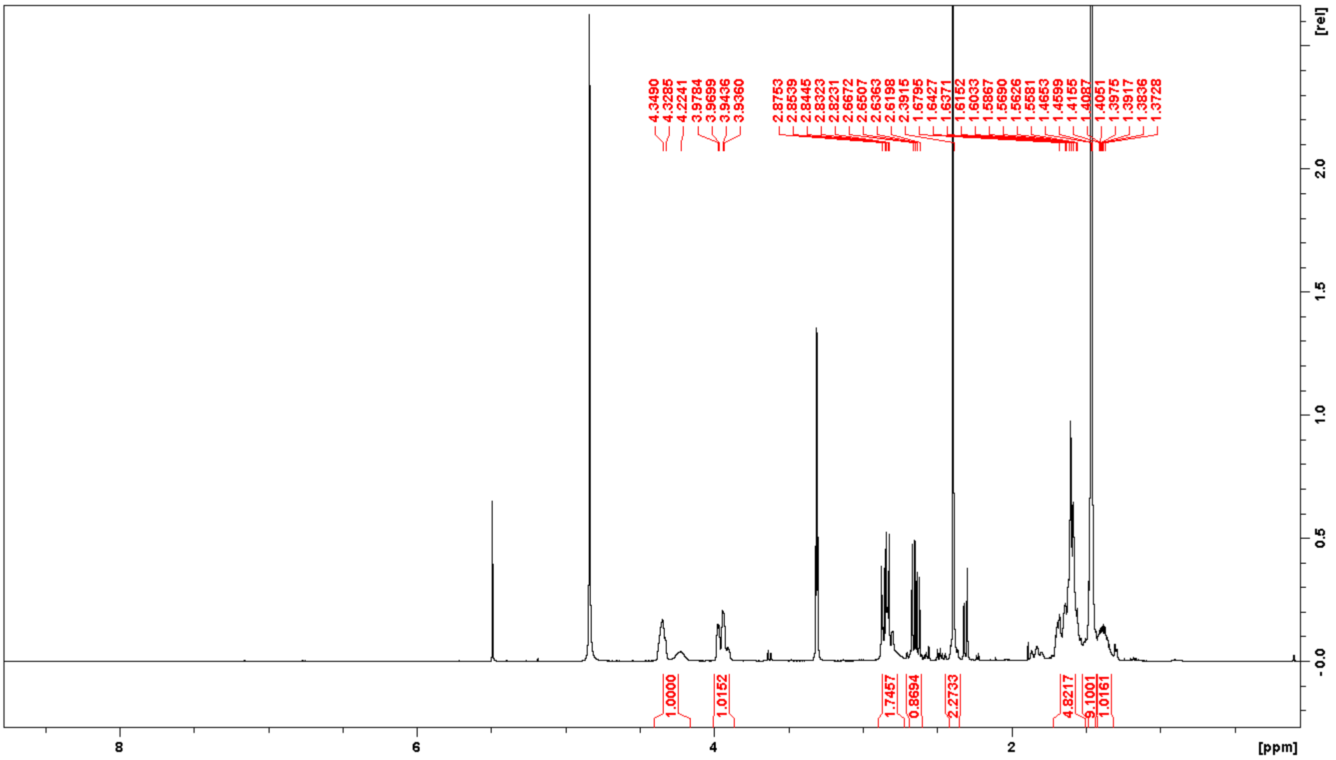

Tert-butyl (S)-3-formylisoxazolidine-2-carboxylate (**7**)

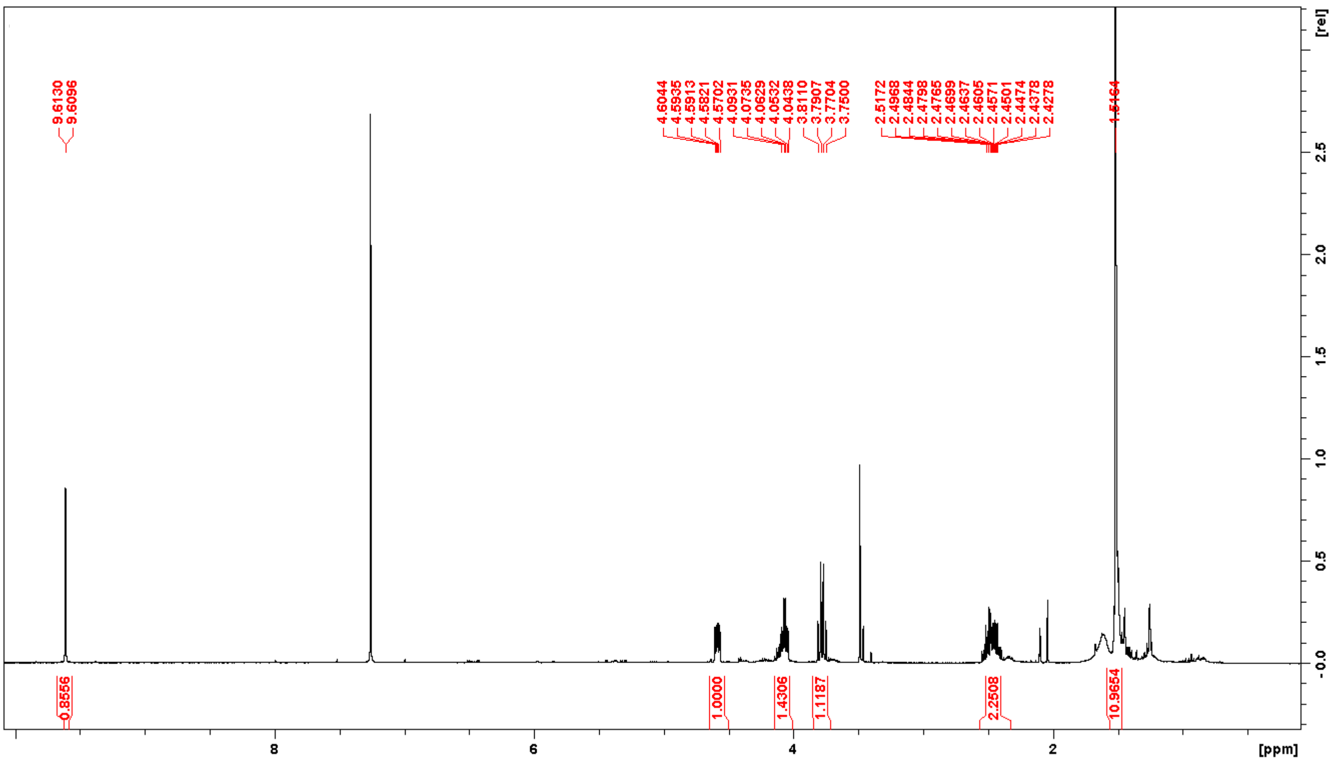

O-acetyl-N-(((S)-2-(tert-butoxycarbonyl)isoxazolidin-3-yl)methyl)-N-methylhydroxylammonium (**8**)

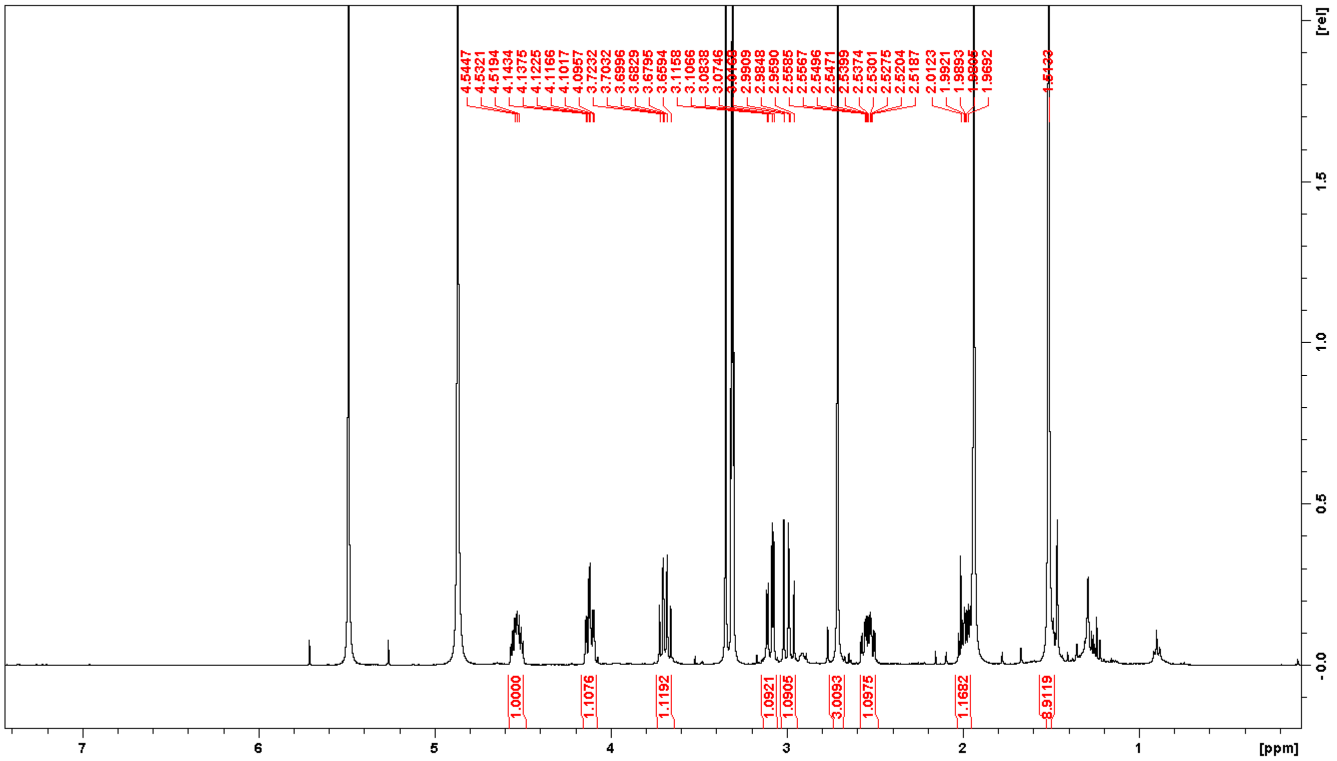

1-(tert-butyl) 2-methyl (2S,4S)-4-(dimethylamino)pyrrolidine-1,2-dicarboxylate (**10**):

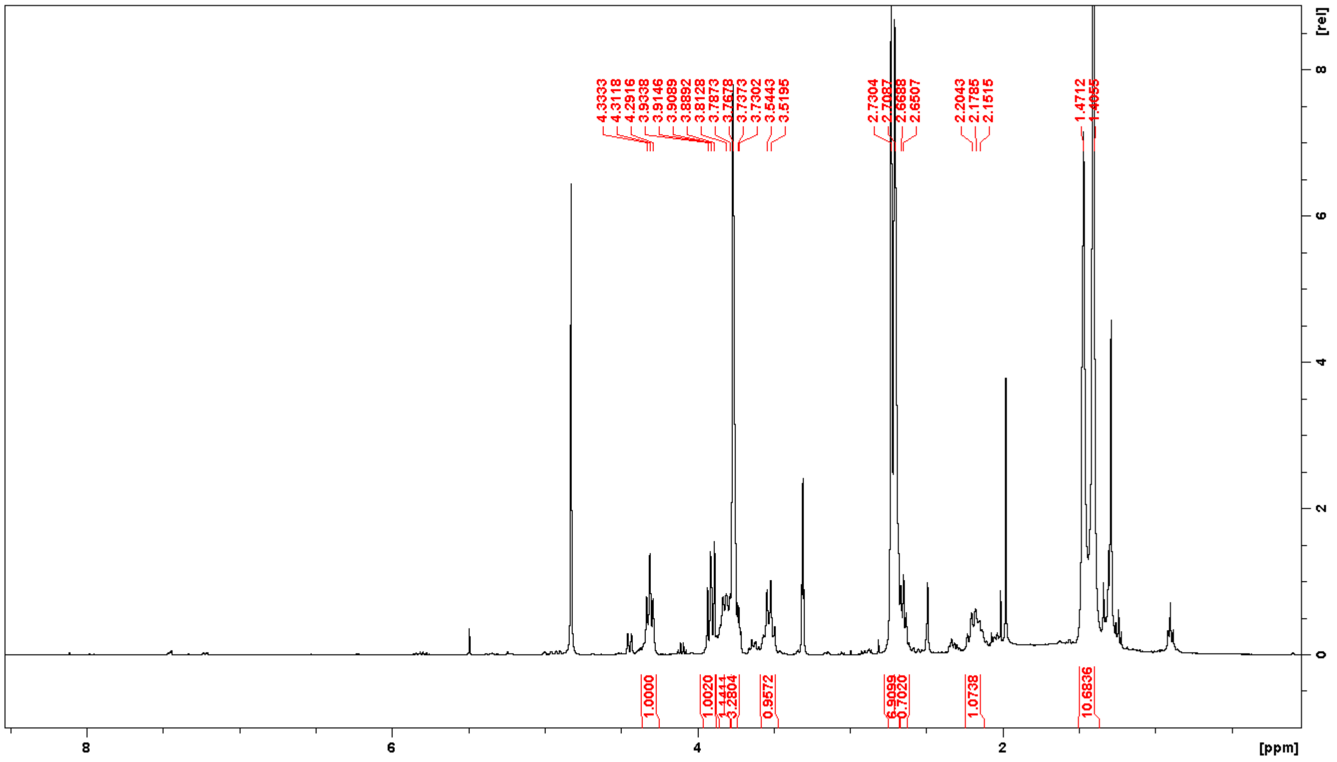

Tert-butyl 2-(((3-(dimethylamino)propyl)amino)methyl)pyrrolidine-1-carboxylate (**13**)

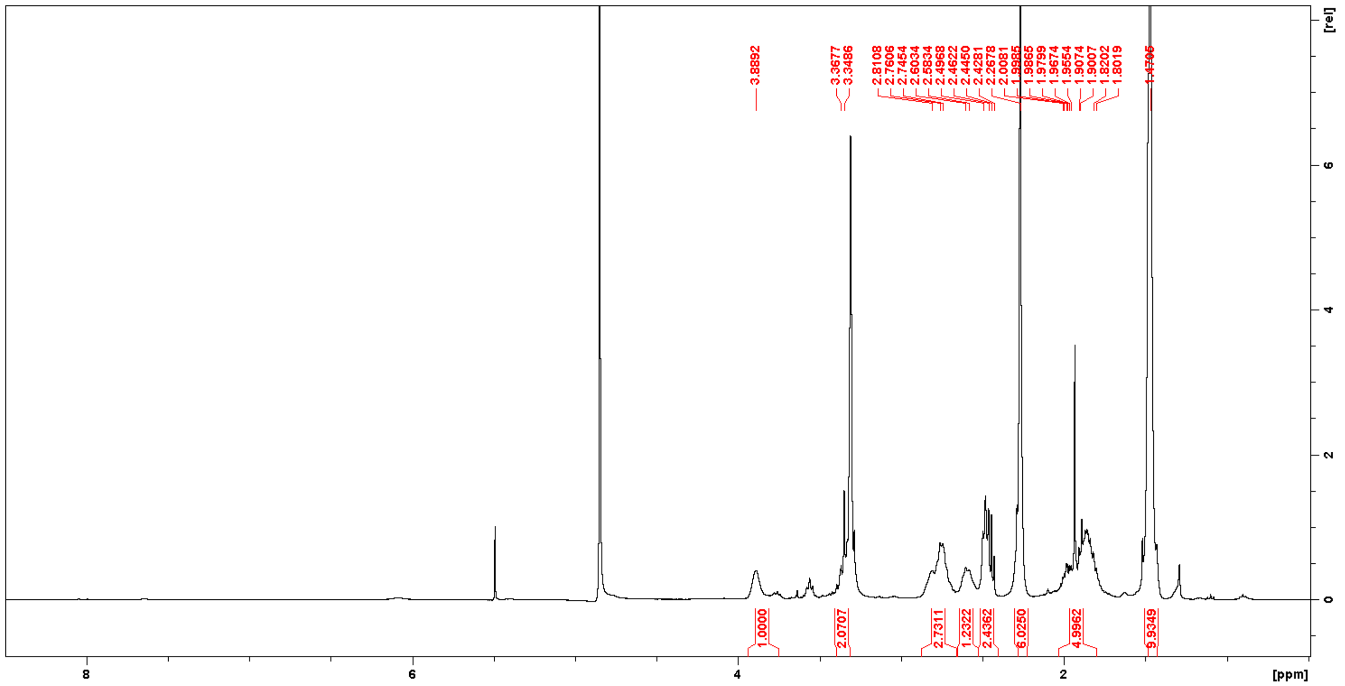

3.8892
3.3677
3.3486
2.8108
2.7606
2.7454
2.6034
2.5834
2.4968
2.4622
2.4450
2.4281
2.2678
2.0081
1.9885
1.9865
1.9799
1.9674
1.9554
1.9074
1.9007
1.8202
1.8019
1.4795
1.0000
2.0707
2.7311
1.2322
2.4362
6.0250
4.9962
9.9349
[rel]
[ppm]

Boc-Hyp(All)-OMe (**15**)

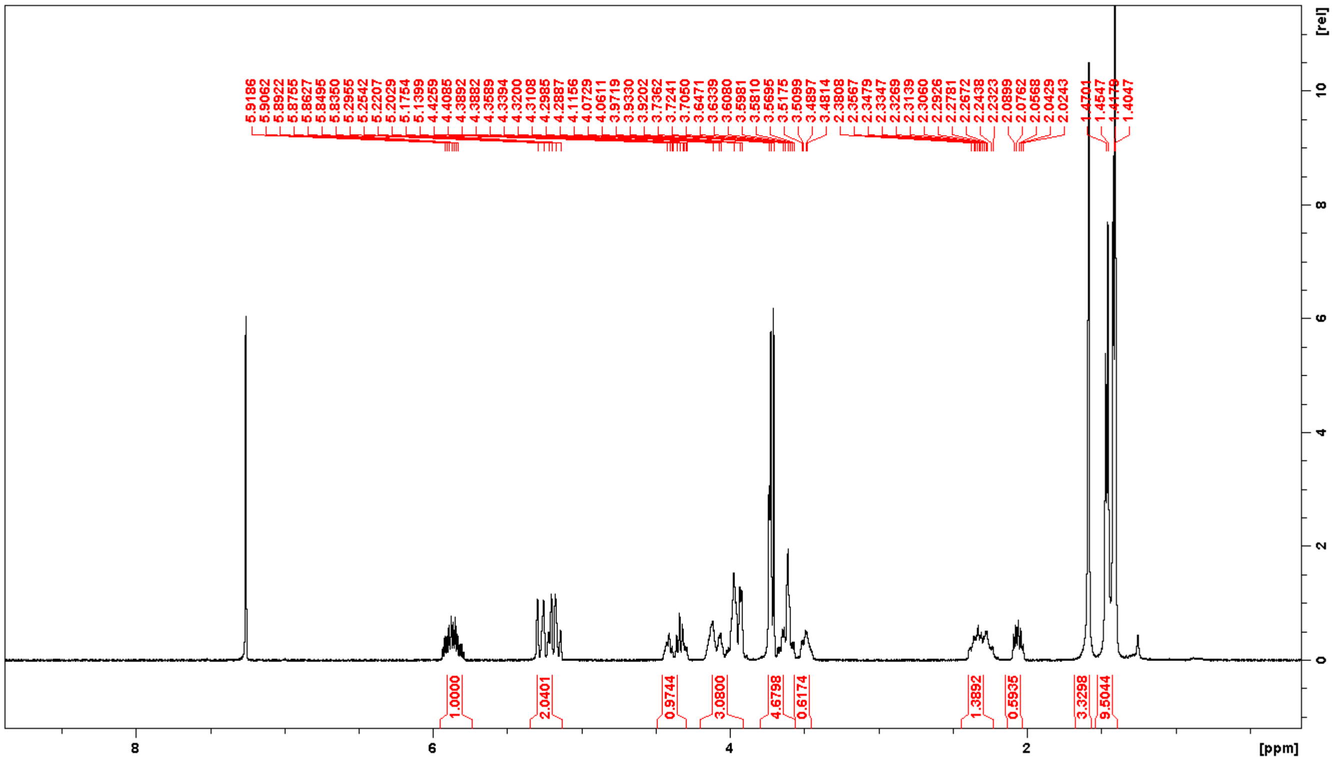

Boc-Hyp(2-hydroxyethoxy)-OMe (**17**)

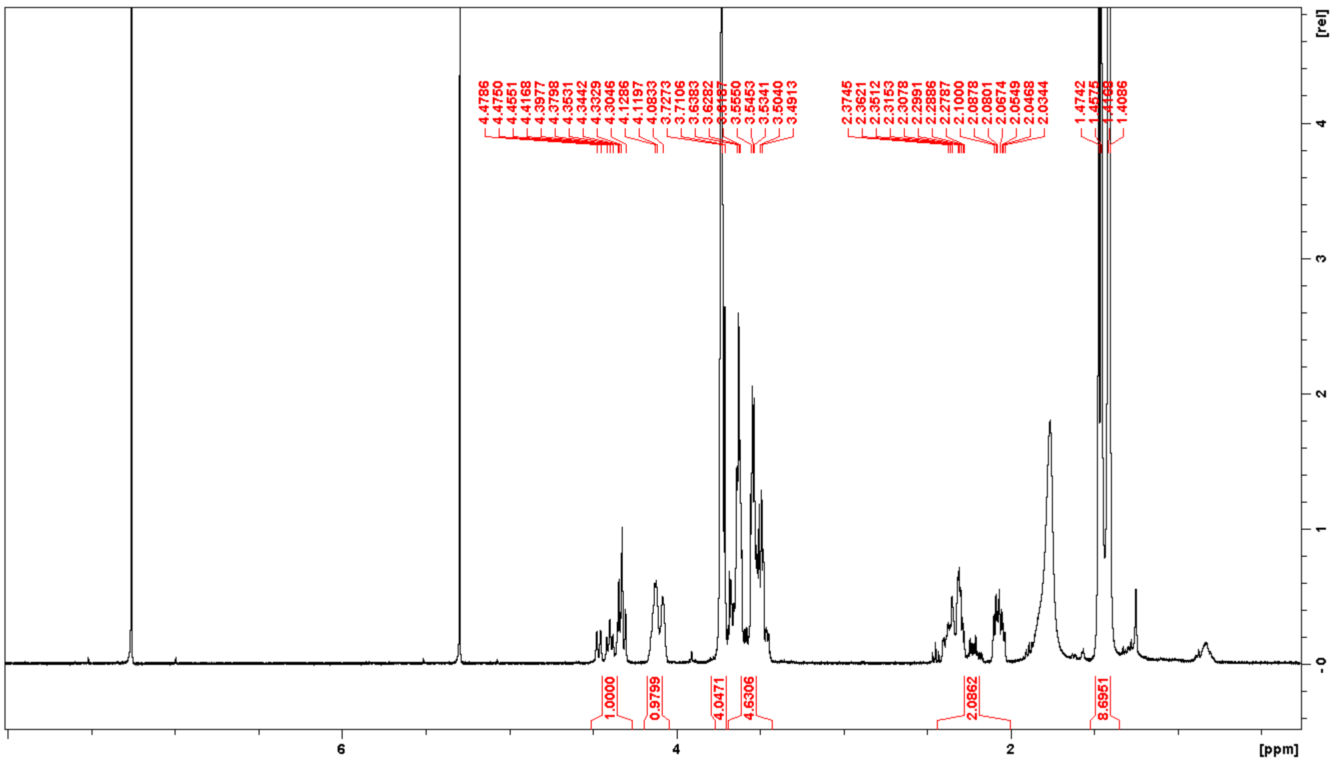

Boc-R848

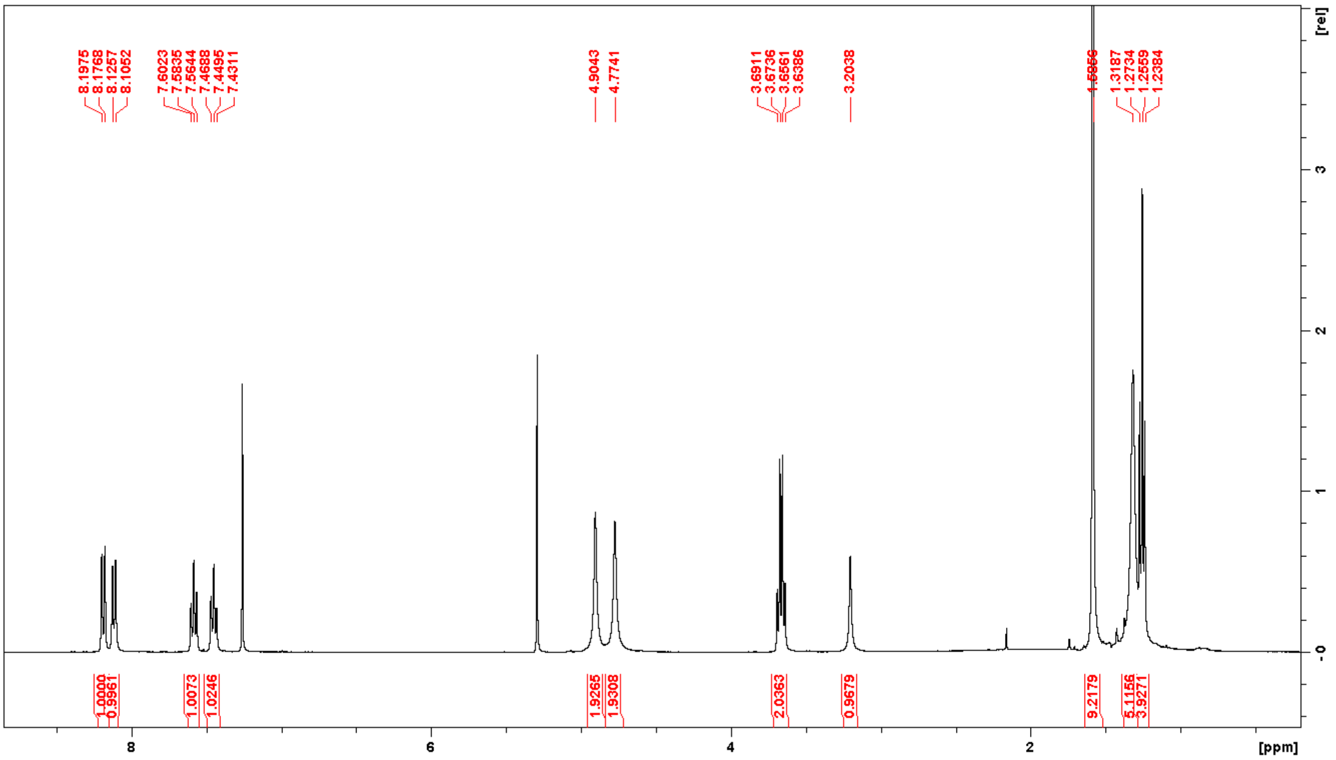
8.1975
8.1768
8.1257
8.1052
7.6023
7.5835
7.5644
7.4688
7.4495
7.4311
4.9043
4.7741
3.6911
3.6736
3.6561
3.6386
3.2038
1.3187
1.2734
1.2559
1.2384
1.0000
0.9961
1.0073
1.0246
1.9265
1.9308
2.0363
0.9679
9.2179
5.1156
3.9271
[rel]
[ppm]

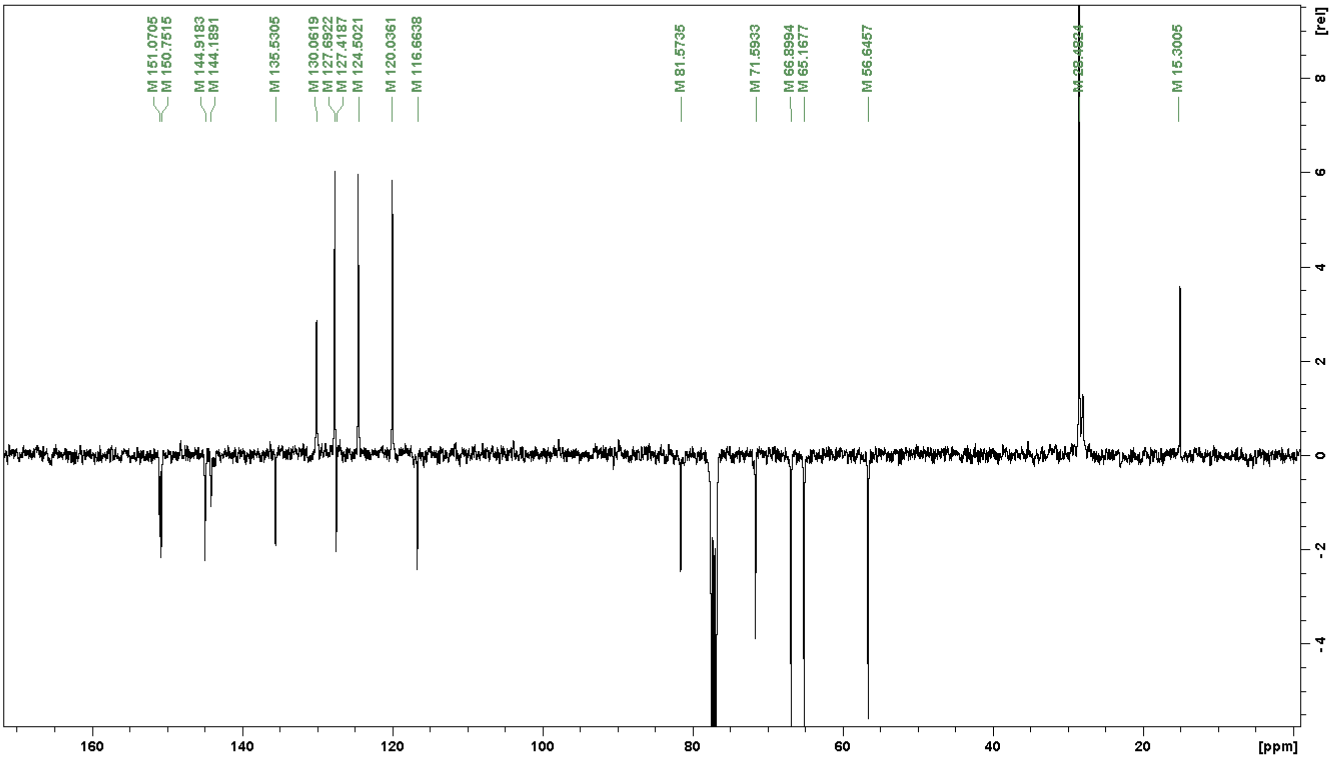
M 151.0705
M 150.7515
M 144.9183
M 144.1891
M 135.5305
M 130.0619
M 127.6922
M 127.4187
M 124.5021
M 120.0361
M 116.6638
M 81.5735
M 71.5933
M 66.8994
M 65.1677
M 56.6457
M 15.3005
[rel]
[ppm]

R848-PNP

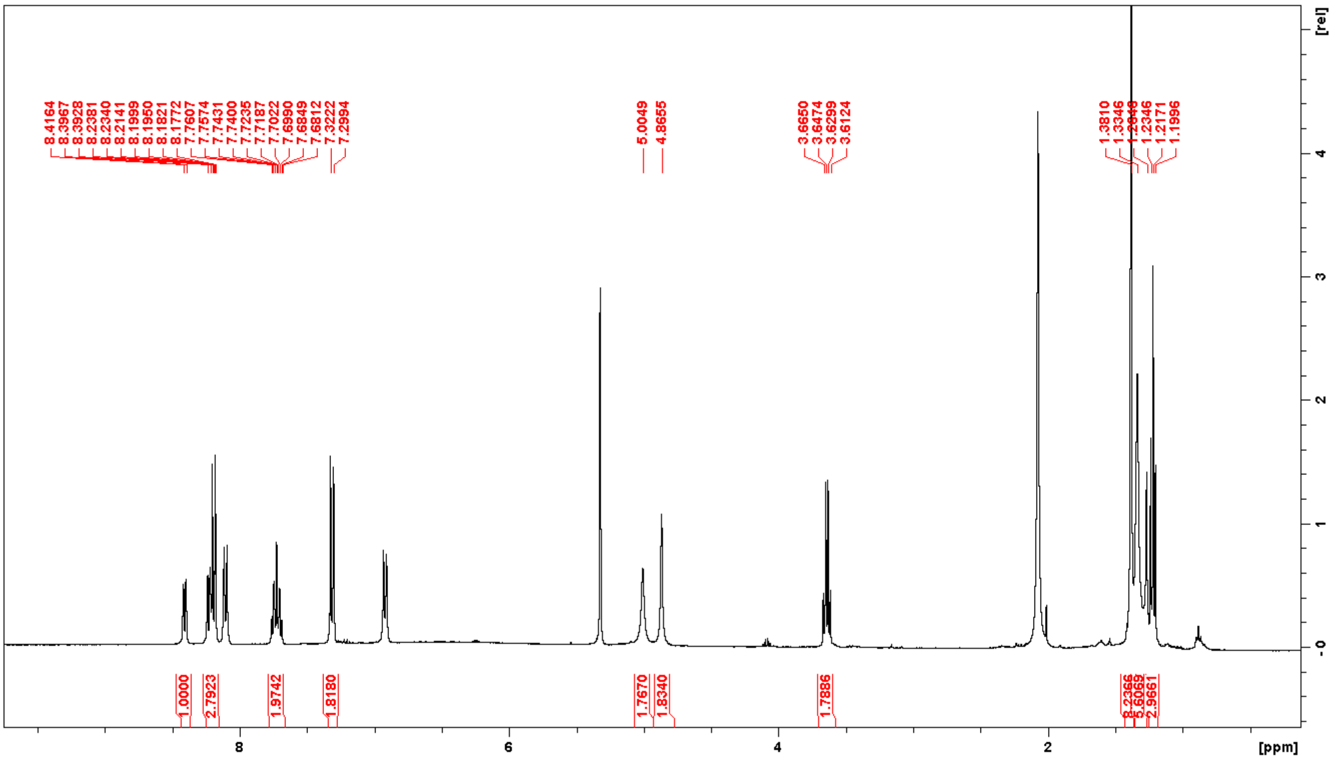

Supplement: Supplementary file 1 — Supporting Information [file CMDC-17-0-s001.pdf]
